# Dose-volume predictors of post-radiation xerostomia in head and neck cancer: A systematic review

**DOI:** 10.1016/j.ctro.2026.101282

**Published:** 2026-09-06

**Authors:** Jikai Zhou, Xuanhe Hou, Wenjie Liang, Frank Hoebers, Jolien Heukelom, Andre Dekker, Cecile Wolfs, Petros Kalendralis

**Affiliations:** a Maastro, Maastricht Radiation Oncology Institute, Maastricht, the Netherlands; b Department of Radiation Oncology, GROW – School for Oncology and Reproduction, Maastricht University Medical Centre+, Maastricht, the Netherlands

**Keywords:** Head and neck cancer, Xerostomia, Radiotherapy, Dose-volume predictors, Parotid gland, Systematic review

## Abstract

**Background and purpose:**

Radiation-induced xerostomia remains a major toxicity after radiotherapy for head and neck cancer (HNC). Although QUANTEC-based parotid dose constraints are widely applied, increasing evidence suggests that additional salivary and oral structures may contribute to xerostomia risk. This systematic review aimed to summarize contemporary evidence on dose-volume predictors of post-radiotherapy xerostomia in HNC.

**Materials and methods:**

This PRISMA-based systematic review was registered in PROSPERO (CRD42024519338). PubMed, Embase, Cochrane Library, and Web of Science were searched for studies published between January 2013 and August 2025. Studies evaluating dose-volume predictors of xerostomia in adult HNC patients treated with curative-intent radiotherapy were included.

**Results:**

Fifty-one studies involving 10,789 patients were included. Most patients received intensity-modulated radiotherapy. Contralateral parotid gland mean dose (Dmean) was the most consistently validated predictor of xerostomia and was supported by moderate-certainty evidence. Multiple studies reported increased risk of moderate-to-severe xerostomia when contralateral parotid Dmean exceeded 24–26 Gy, whereas Dmean below 20 Gy was associated with better salivary preservation. Bilateral parotid Dmean around 26 Gy was also reported. However, support for specific numerical planning thresholds remained limited. Dosimetric parameters of the submandibular glands and oral cavity were also independently associated with xerostomia risk. Emerging evidence suggested potential roles of parotid substructures, including ducts and stem cell-rich regions.

**Conclusion:**

Parotid gland mean dose remains the most robust predictor of post-radiotherapy xerostomia in HNC. Additional dosimetric information from the submandibular glands, oral cavity, and parotid substructures may further improve xerostomia risk stratification and support future NTCP modeling.

## Highlights

• Contemporary evidence on xerostomia predictors after head and neck RT.
• Contralateral parotid mean dose showed the strongest evidence.
• Submandibular and oral cavity doses were associated with xerostomia.
• Multiple salivary structures may improve NTCP modeling.
• Findings support a multi-organ approach to xerostomia prevention.

## Graphical abstract

**Figure:**
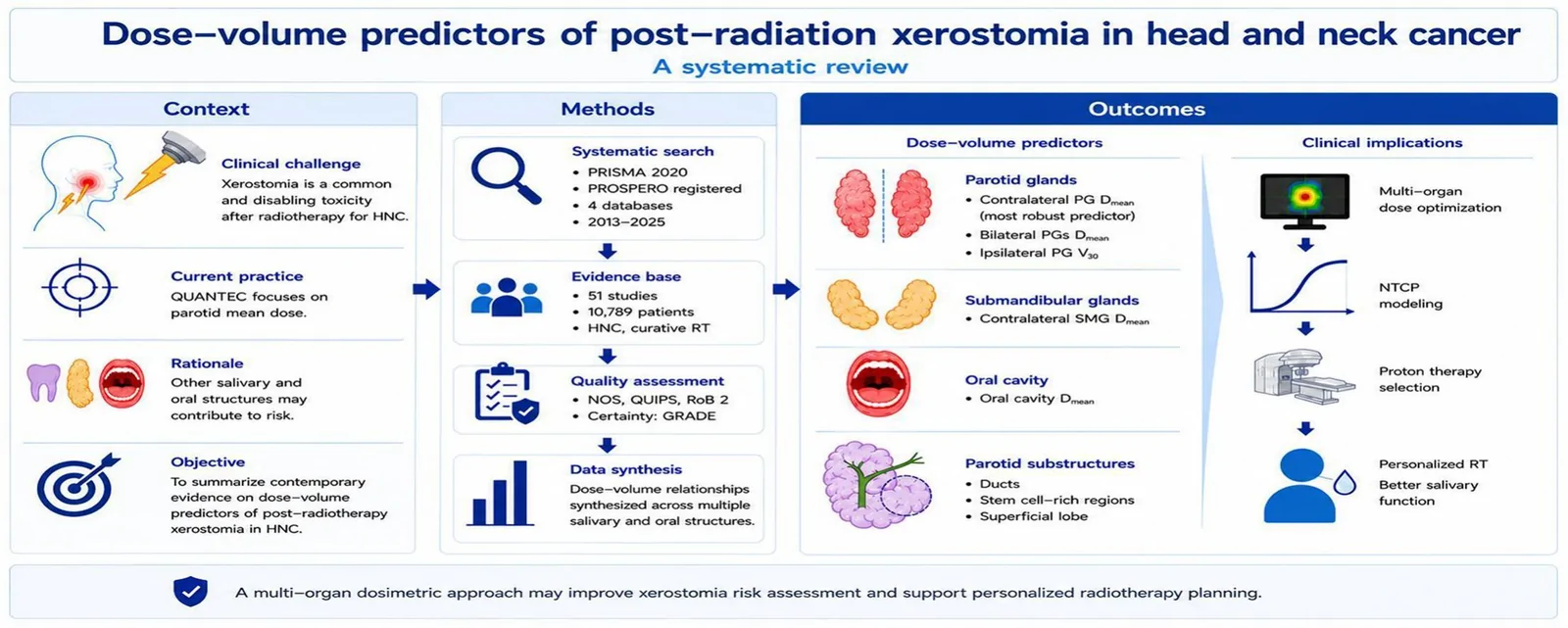

## 1 Introduction

Head and neck cancer (HNC), encompassing malignancies arising in the oral cavity, pharynx, larynx, and other related sites, ranks among the ten most common cancers worldwide, with approximately 800,000 new cases annually, accounting for about 4–5% of all cancer diagnoses and representing a major global public health burden [1]. Currently, approximately 75% of patients with head and neck squamous cell carcinoma (HNSCC) will benefit from radiotherapy at some point during their course of treatment [2]. However, radiotherapy is associated with both acute and late toxicities. Among these, xerostomia is one of the most common and disabling radiation-induced toxicities during and after treatment, with substantial and lasting impacts on oral health and quality of life [3]. Therefore, minimizing clinically significant xerostomia remains a priority. In clinical practice, prevention is commonly guided by the QUANTEC (Quantitative Analyses of Normal Tissue Effects in the Clinic) consensus, which identifies parotid mean dose (Dmean) as a robust predictor and recommends keeping Dmean <20 Gy to at least one parotid or < 25 Gy to both glands [4].

However, several limitations of relying solely on this consensus have become evident in contemporary radiotherapy practice. First, the threshold-based nature of the QUANTEC recommendations may not fully capture the continuous dose–response relationship between salivary gland irradiation and xerostomia risk observed in contemporary radiotherapy studies, including evidence from dose–response analyses and normal tissue complication probability (NTCP) modeling [5], [6]. Second, increasing evidence suggests that xerostomia risk is influenced not only by the parotid glands but also by the submandibular glands, oral cavity, and other functionally relevant salivary structures [7], [8], [9], [10]. Third, the QUANTEC recommendations were primarily derived from earlier-generation radiotherapy techniques and reflect a narrative expert consensus rather than a systematic synthesis of contemporary evidence. With the widespread adoption of modern Intensity Modulated Radio Therapy (IMRT) techniques and substantial new data generated over the past decade, the evidence landscape underlying xerostomia risk has evolved considerably.

Therefore, an updated systematic synthesis of contemporary evidence is warranted. The present review aimed to summarize dose–volume predictors of post-radiotherapy xerostomia in patients with head and neck cancer, evaluate the consistency and certainty of the available evidence, summarize clinically relevant planning thresholds, and discuss the implications for NTCP modeling and future function-preserving radiotherapy strategies.

## 2 Materials and methods

This systematic review was conducted in accordance with the Preferred Reporting Items for Systematic Reviews and Meta-Analyses (PRISMA) 2020 guidelines [11]. The review protocol was prospectively registered in PROSPERO (CRD42024519338).

### 2.1 Search strategy

We systematically searched four databases-PubMed, Embase, the Cochrane Library, and Web of Science-for articles published from January 1, 2013 to August 22, 2025, with the last search conducted on Aug 22, 2025. The starting date of January 1, 2013 was chosen to align with the publication of the last major systematic review addressing xerostomia dose–volume relationships [12], thereby allowing the present review to provide an updated synthesis of evidence generated in the modern IMRT era. The search strategy was developed and conducted under the guidance of two Medical Information Specialists at Maastricht University. In brief, the search strategy was structured around four core concepts: [1] head and neck cancer, [2] radiotherapy, [3] xerostomia, and [4] radiation dosage. For each database, these concepts were combined using Boolean operators and implemented through a combination of controlled vocabulary (e.g., MeSH terms in PubMed and Cochrane, Emtree terms in Embase) and free-text keywords, including synonyms and spelling variants. Database-specific filters were applied to exclude non-original publications (e.g., reviews, editorials, conference abstracts) and non-human studies. The full, database-specific search strings and syntax are provided in Supplementary Table 1. After deduplication, two authors (JZ and XH) independently screened the titles and abstracts of all records to assess their relevance to the study objectives. Any discrepancies were resolved through discussion.

### 2.2 Eligibility criteria

Studies were selected according to the PICOS framework. Eligible studies met the following criteria: [1] population: adult patients (≥18 years) diagnosed with head and neck cancer treated with curative-intent radiotherapy, either definitive or postoperative (adjuvant); [2] intervention/exposure: radiotherapy with or without chemotherapy; [3] comparison: different dose–volume parameters or exposure groups; [4] outcomes: post-radiation xerostomia (incidence or severity); and [5] study design: original studies reporting dose–volume predictors of xerostomia.

Studies were excluded if they were [1] non-original articles (e.g., editorials, conference proceedings, review articles, or case reports), [2] non-English publications, or [3] did not include dose–volume analysis relevant to xerostomia.

### 2.3 Data extraction

A standardized form was developed for data extraction. All included studies reported dose–volume analyses related to xerostomia. The following data were extracted from each eligible study:

(1) Bibliographic information;
(2) Patient characteristics, including tumour type, radiotherapy technique, and use of chemotherapy;
(3) Information on post-radiotherapy xerostomia, including assessment time points, assessment methods;
(4) Main study outcomes, including reported dose–volume predictive parameters, cutoff values, relative effect for xerostomia risk.

Effect size measures (e.g., ORs or HRs) were extracted as reported in the original studies without conversion to a standardized metric, as no meta-analysis was performed.

Surgical status (definitive versus postoperative radiotherapy) was not systematically extracted, as it was inconsistently reported across studies and the primary focus of this review was on post-radiotherapy dosimetric predictors of xerostomia.

### 2.4 Study classification

To facilitate evidence synthesis, ensure methodological consistency, and identify studies potentially suitable for quantitative synthesis, the included studies were categorized into three levels of evidence based on the statistical robustness and interpretability of their reported dose–response analyses. Level A comprised studies reporting multivariable (adjusted) dose–response models that met at least one of the following criteria: (a) provision of adjusted odds ratios (ORs) or hazard ratios (HRs) for continuous dose exposure (e.g., per 1 Gy or per 1%); or (b) reporting of predefined or data-driven dose thresholds (e.g., Youden index–based) accompanied by adjusted effect estimates. These studies were prioritized because they provide quantitatively interpretable and independent dose–response estimates while accounting for potential confounders, and therefore represent the only studies potentially suitable for assessing the feasibility of quantitative synthesis.

Level B included studies that performed discriminative or threshold-based analyses without reporting adjusted effect estimates, such as receiver operating characteristic (ROC) analysis with area under the curve (AUC), Youden index–derived thresholds without accompanying adjusted measures, or other performance metrics (e.g., sensitivity/specificity, PPV/NPV). These analyses demonstrate predictive performance but do not provide directly comparable dose–response parameters.

Level C comprised studies limited to univariate or exploratory analyses, including simple statistical comparisons such as *t*-tests, χ^2^ tests, Mann–Whitney *U* tests or correlation analyses, without multivariable modeling.

If a study employed multiple analytical approaches, it was classified according to the highest level fulfilled. Data extraction and evidence grading were performed independently by two reviewers (JZ and XH), with discrepancies resolved through discussion and adjudication by a third reviewer (WL).

This grading framework was used to support structured evidence synthesis, with Level A studies informing dose–response interpretation, Level B studies providing complementary discriminative evidence, and Level C studies offering contextual insights.

### 2.5 Quality assessment

Given the heterogeneity of the included studies in terms of research objectives and design, we predefined (a priori) the assignment of risk-of-bias assessment tools according to study design characteristics and study intent to ensure methodological consistency and comparability of results. Specifically:

Randomized controlled trials (RCTs): assessed using the Cochrane Risk of Bias 2 (RoB 2) tool;

Observational cohort studies (prospective or retrospective, including non-randomized comparative studies with historical controls): assessed using the Newcastle–Ottawa Scale (NOS);

Studies primarily aimed at prognostic factor evaluation or model development (e.g., NTCP/dose–response modeling, predictive/nomogram analyses, or pooled/ancillary analyses derived from single-arm trials or registries): assessed using the Quality In Prognostic Studies (QUIPS) tool.

The selection of QUIPS was based on its suitability for prognostic research, as it allows for evaluation of key domains such as study participation, attrition, confounding control, statistical modeling, and reporting-areas that are insufficiently addressed within the NOS framework.

Quality assessment was performed independently by two reviewers (JZ and XH), and discrepancies were resolved through discussion with a third reviewer (WL).

To achieve consistency across assessment tools, overall study quality was categorized into three levels: high, moderate, and low quality. For the NOS, scores ≥7 were rated as high quality, 5–6 as moderate quality, and ≤ 4 as low quality. For QUIPS, studies with low risk in all domains were rated as high quality, those with one or more moderate-risk domain and no high-risk domains as moderate quality, and those with any high-risk domain as low quality. For RoB 2, studies with low risk, some concerns, and high risk were classified as high, moderate, and low quality, respectively.

### 2.6 Assessment of certainty of evidence

The certainty of evidence was assessed using the GRADE (Grading of Recommendations Assessment, Development and Evaluation) framework [13]. GRADE assessments were performed for bodies of evidence comprising studies evaluating the same anatomical structure and comparable dosimetric parameter in relation to xerostomia. Evidence certainty was evaluated across the domains of study limitations, inconsistency, indirectness, imprecision, and other considerations. Observational evidence was initially considered low certainty and subsequently downgraded or upgraded according to the GRADE framework, where appropriate.

Numerical thresholds identified during the narrative synthesis were grouped according to the corresponding organ–dose metric combination and evaluated separately for threshold-specific certainty using the same GRADE domains. Continuous dose–response studies were considered indirect supportive evidence when they did not directly evaluate the proposed numerical threshold. The evidence assessments were performed independently by two reviewers (JZ and XH), with discrepancies resolved through discussion with a third reviewer (WL).

## 3 Results

### 3.1 Study selection

**Fig. 1:**
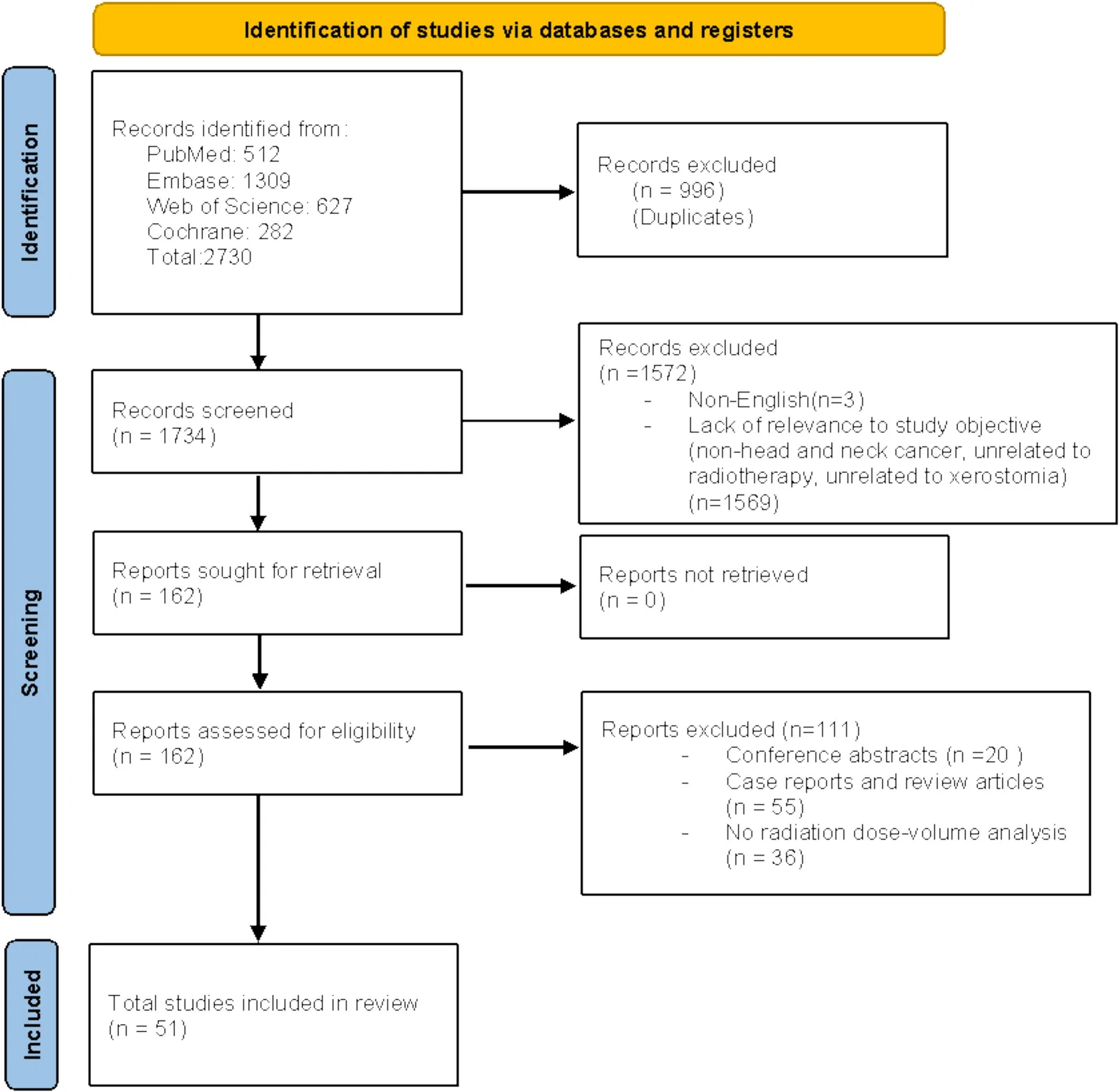
The preferred reporting items for systematic reviews and meta-analysis (PRISMA) 2020 flow diagram.

The search process is summarized in Fig. 1. A total of 2730 articles were initially identified through the database search.

In the first screening round, duplicate records were removed (*n* = 996). In the second round, non-English articles (*n* = 3) and studies irrelevant to the topic of this systematic review (*n* = 1569) were excluded. In the third round, no articles were excluded due to inaccessible full texts (*n* = 0). In the fourth round, conference abstracts (*n* = 20), case reports and review articles (*n* = 55), and studies without radiotherapy dose–volume analysis (*n* = 36) were excluded. Ultimately, 51 studies were included in the final analysis.

Supplementary Table 2 provides a detailed summary of key study characteristics for all 51 included publications, including first author and year, sample size, tumour type, radiotherapy technique, chemotherapy use, timing of xerostomia assessment, xerostomia assessment methods, dosimetric parameters, cutoff values, relative effect, and statistical methods level.

### 3.2 Study characteristics

#### 3.2.1 Study design

Based on study design, 43 of 51 studies (84.3%) were cohort studies, 2 of 51 (3.9%) were RCTs, and the remaining 6 of 51 (11.8%) employed other designs, including cross-sectional studies, case series, and secondary analyses of data from single-arm trials.

According to data collection methods, 31 of 51 studies (60.8%) were retrospective, 19 of 51 (37.3%) were prospective (including RCTs and post-hoc/pooled/ancillary analyses derived from prospective single-arm trials), and 1 of 51 (2.0%) used a mixed design.

#### 3.2.2 Outcome measures

**Fig. 2:**
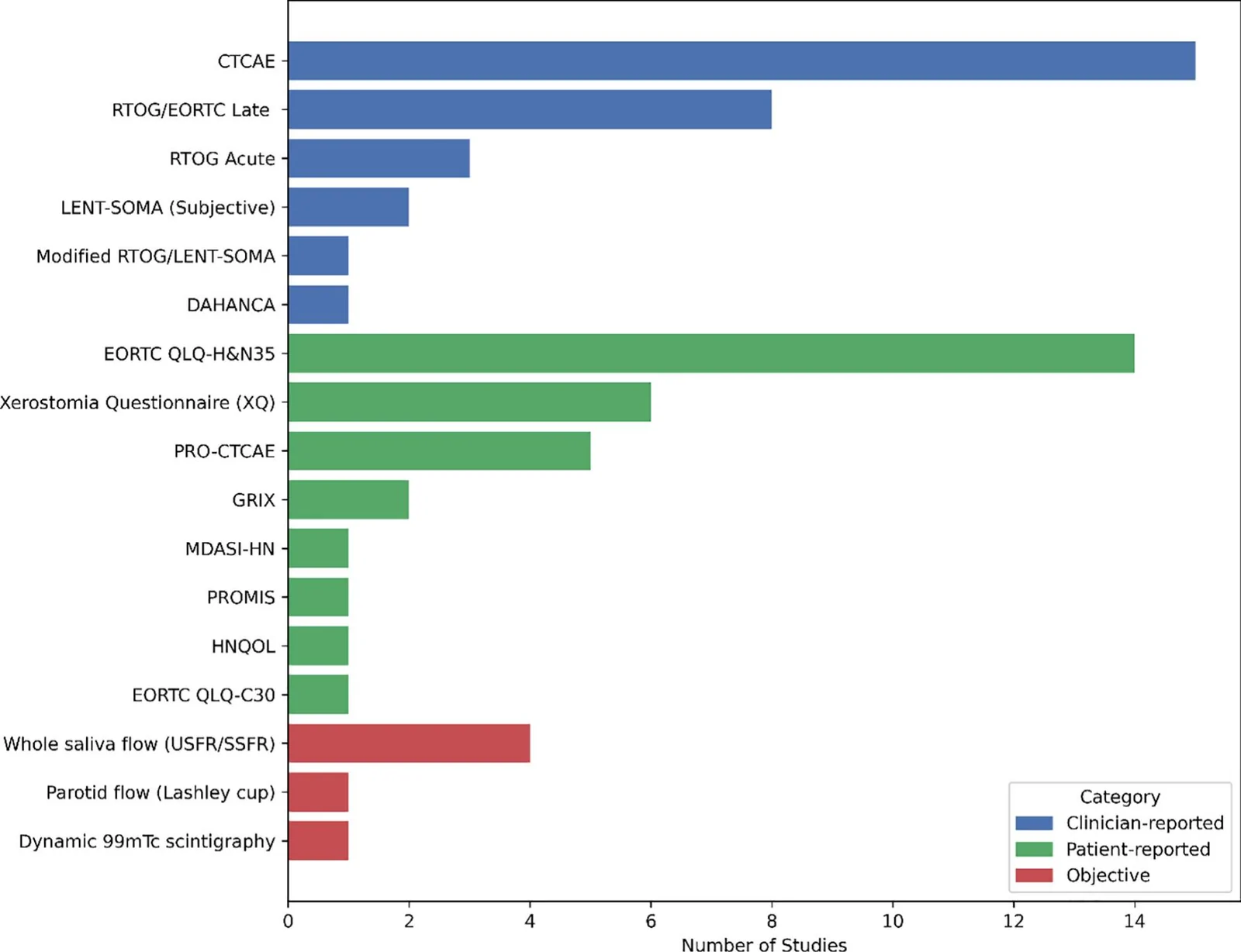
Distribution of xerostomia assessment tools. Abbreviations: CTCAE, common terminology criteria for adverse events; EORTC QLQ-H&N35, EORTC quality of life questionnaire–head and neck module; LENT-SOMA, late effects in Normal tissues–subjective, objective, management, analytic; PRO-CTCAE, patient-reported outcomes version of CTCAE; USFR, unstimulated salivary flow rate, SSFR, stimulated salivary flow rate; Lashley cup, device for collecting parotid salivary flow; 99mTc, technetium-99 m. Colors: Blue = clinician-reported; green = patient-reported; red = objective

The assessment methods for xerostomia in the included studies were categorized into three main types: clinician-reported, patient-reported, and objective quantitative evaluations (Fig. 2).

Among clinician-reported tools, the most frequently used was the Common Terminology Criteria for Adverse Events (CTCAE) (15 studies), followed by the Radiation Therapy Oncology Group/European Organization for Research and Treatment of Cancer (RTOG/EORTC) Late Radiation Morbidity Scoring System (8 studies) and the RTOG Acute Toxicity Criteria (3 studies). A few studies used the Late Effects in Normal Tissues–Subjective, Objective, Management, Analytic (LENT-SOMA) system, its modified versions (e.g., modified RTOG/LENT-SOMA), or criteria developed by the Danish Head and Neck Cancer Group (DAHANCA).

For patient-reported outcomes, the most commonly used instrument was the European Organization for Research and Treatment of Cancer Quality of Life Questionnaire–Head and Neck Module (EORTC QLQ-H&N35) (14 studies), followed by the Xerostomia Questionnaire (XQ) (6 studies) and the Patient-Reported Outcomes version of the CTCAE (PRO-CTCAE) (5 studies). Other instruments occasionally employed included the Groningen Radiotherapy-Induced Xerostomia Questionnaire (GRIX) (2 studies), the MD Anderson Symptom Inventory–Head and Neck Module (MDASI-HN), the Patient-Reported Outcomes Measurement Information System (PROMIS), and the Head and Neck Quality of Life Questionnaire (HNQOL).

Objective assessment methods were less frequently used. A total of 4 studies employed whole saliva flow measurements (USFR and/or SSFR), while parotid duct flow quantification using Lashley cups and dynamic 99mTc salivary gland scintigraphy were each reported in 1 study.

Overall, CTCAE and EORTC QLQ-H&N35 were the most frequently applied clinician-reported and patient-reported assessment tools, respectively.

#### 3.2.3 Timing of assessment

**Fig. 3:**
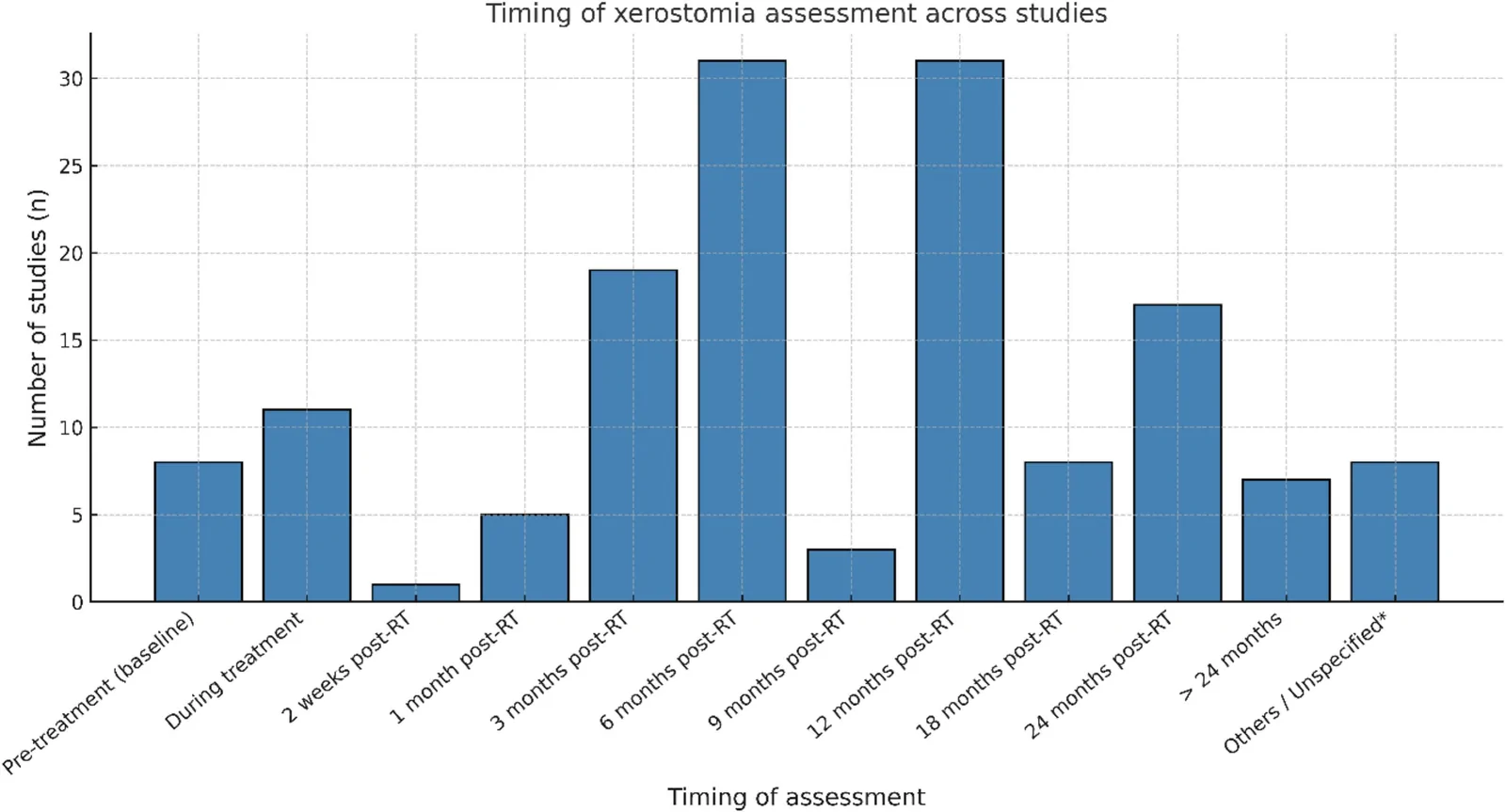
Timing of xerostomia assessment across studies. Note: * Includes studies reporting peak-late toxicity windows, ≥6-month unspecified, first assessment between 9 and 24 months, author-defined intervals (<6 / 6–11 / 12–24 months), most recent questionnaire only, or not reported (*n* = 8).

The timing of xerostomia assessment varied across the included studies (Fig. 3), and most studies reported more than one assessment time point. The most frequently used evaluation time points were 6 months and 12 months after radiotherapy (each *n* = 31/51, 60.8%), followed by 3 months (*n* = 19/51, 37.3%) and 24 months (*n* = 17/51, 33.3%). Assessments conducted before treatment (baseline) and during treatment were reported in *n* = 8/51 (15.7%) and *n* = 11/51 (21.6%) studies, respectively. Additionally, *n* = 8/51 (15.7%) studies did not specify a precise assessment time point. These studies used alternative or unclear definitions, such as reporting the peak value within 6 weeks to 24 months, the first questionnaire administered between 9 and 24 months, custom intervals (<6 months, 6–11 months, 12–24 months), or only the most recent assessment, while some did not report timing details at all. Such studies were collectively categorized as having “other/unspecified” assessment timing.

According to the timing of occurrence, xerostomia is generally classified into acute xerostomia (≤3 months after radiotherapy) and late xerostomia (>3 months) [2]. Among the 41 studies with clearly defined assessment time points, 24 of 41 (58.5%) investigated acute post-radiotherapy xerostomia, and 34 of 41 (82.9%) examined late xerostomia. Specifically, 7 of 41 (17.1%) focused exclusively on acute xerostomia, 17 of 41 (41.5%) focused solely on late xerostomia, and 17 of 41 (41.5%) included both acute and late phases.

#### 3.2.4 Study quality and risk of bias

In accordance with the predefined protocol described in the Methods section, 41 studies were assessed using NOS, 8 studies using QUIPS, and 2 studies using RoB 2.

Based on the unified grading criteria, the overall quality distribution was as follows: high quality in 17 of 51 studies (33.3%), moderate quality in 26 of 51 (51.0%), and low quality in 8 of 51 (15.7%).

The number and proportion of high-, moderate-, and low-quality studies stratified by assessment tool are summarized in Table 1. The study-specific grading results for the three different quality assessment methods are presented in Supplementary Tables 3,4, and 5, and a harmonized summary of study-level quality assessments across all included studies is provided in Supplementary Table 6.

**Table 1:** Summary of quality assessment tools and overall quality distribution.

Tool	No. of studies	Study type	Quality classification rule	High quality/Low risk of bias	Moderate quality/Some concerns	Low quality /High risk of bias
NOS	41	Observational	≥7 = High quality; 5–6 = Moderate quality; ≤4 = Low quality	17	18	6
QUIPS	8	Prognostic / NTCP	All domains low risk = Low risk of bias; ≥1 domain moderate & none high = Some concerns; ≥1 domain high = High risk of bias	0	6	2
RoB 2	2	RCT	Low risk of bias = Low risk of bias; Some concerns = Some concerns; High risk of bias = High risk of bias	0	2	0

Note: NOS evaluates study quality (high = better quality), while QUIPS and RoB 2 assess risk of bias (high = higher risk).

To ensure consistency, results are presented as High quality / Low risk of bias, Moderate quality / Some concerns, and Low quality / High risk of bias.

#### 3.2.5 Patient characteristics

A total of 10,789 participants were included across 51 studies, with the sample size of individual studies ranging from 30 to 1145 (median: 121 participants).

#### 3.2.6 Dosimetry

##### 3.2.6.1 Type of radiotherapy

Most studies (49 of 51, 96%) reported the type of radiotherapy used. No study exclusively enrolled patients treated with two-dimensional radiotherapy (2D-RT). One study exclusively used three-dimensional conformal radiotherapy (3D-CRT), whereas 42 studies exclusively enrolled patients treated with intensity-modulated radiotherapy (IMRT). An additional 6 studies included mixed cohorts treated with both 3D-CRT and IMRT-based techniques, and 2 studies did not report on the radiotherapy technique. Among studies that included IMRT-based techniques, 15 reported the use of volumetric modulated arc therapy (VMAT) and/or helical tomotherapy (HT). Two studies also included patients treated with intensity-modulated proton therapy (IMPT).

##### 3.2.6.2 Summary of dosimetric findings

The structures and dosimetric parameters associated with post-radiotherapy xerostomia identified in the included studies are summarized in Table 2. To enhance readability and emphasize clinically relevant findings, Table 2 presents the most frequently reported dosimetric parameters for each organ at risk, while the complete set of reported parameters is provided in Supplementary Table 7.

**Table 2:** Key dosimetric predictors of post-radiation xerostomia across organs at risk.

Organ at risk	Key dosimetric parameter (No. of studies)	No. of studies reporting this OAR
**Parotid glands (PG)**		
Contralateral PG	Dmean (16)	20
Ipsilateral PG	V30 (4), Dmean (3)	8
Bilateral PGs	Dmean (12)	15
Left/Right PG	Dmean (2)	2

**Submandibular glands(SMGs)**		
Contralateral SMG	Dmean (7)	10
Ipsilateral SMG	Dmean (2)	2
Bilateral SMGs	Dmean (2)	2

**Oral cavity and related structures**		
Oral cavity (OC)	Dmean (9)	12
Floor of mouth (FOM)	Dmean (1)	2
Buccal mucosa (BM)	ln(Dmean Lt + Rt BM) (1)	1

**Other structures**		
Parotid gland–related structures (ducts, superficial PGs, SCR, APG)	Dmean	1–3
Combined Contra glands	Dmean (1)	2
Sublingual glands	Dmean	1
Soft palate	EQ.D2 Dmean	1
Tubarial glands	Dmean	1

Abbreviations: PG, parotid gland; SMG, submandibular gland; OC, oral cavity; FOM, floor of mouth; BM, buccal mucosa; SCR, stem cell region; APG, accessory parotid gland; Dmean, mean dose; Vx, percentage of organ volume receiving ≥x Gy; EQD2, equivalent dose in 2-Gy fractions; Contra, contralateral; Ipsi, ipsilateral; Bilat, bilateral; Lt/Rt, left/right.

Notes: Numbers in parentheses indicate the number of studies reporting each dosimetric parameter. “No. of studies reporting this OAR” refers to the number of unique, non-duplicated studies in which at least one dosimetric parameter for the corresponding organ at risk was reported. For categories summarizing multiple related structures (e.g., parotid gland–related structures), the range of study numbers reflects variation across individual components within that category.

Among the 51 included studies, the parotid glands were the most frequently evaluated organs at risk associated with post-radiation xerostomia. Dosimetric parameters of the contralateral parotid gland were reported in 20 studies, while bilateral parotid parameters were reported in 15 studies. Dmean was the most commonly used dosimetric descriptor, reported for the contralateral parotid gland in 16 studies and for the bilateral parotid glands in 12 studies. In contrast, among the eight studies evaluating ipsilateral parotid dosimetry, volume-based parameters at V30 were reported more frequently (4 studies) than Dmean (3 studies).

In addition to the parotid glands, the submandibular glands (SMGs) and oral cavity were the most commonly investigated structures. Oral cavity–related dosimetric parameters were reported in 12 studies, whereas contralateral SMG parameters were reported in 10 studies, with ipsilateral and bilateral SMG parameters each reported in two studies. Across these structures, reported metrics were predominantly Dmean, while Vx and Dx% parameters were explored only in a limited number of studies. A small number of studies additionally evaluated other potentially relevant structures, including the superficial parotid gland, parotid ducts, and stem cell regions, as well as the floor of mouth, buccal mucosa, sublingual glands, soft palate, and tubarial glands; these structures were each reported in only one to two studies.

Among the dosimetric predictors summarized in Table 2, contralateral parotid mean dose was the most frequently reported, being evaluated in 16 studies. Of these, seven studies met the predefined Level A criteria and were therefore considered potentially eligible for quantitative synthesis. However, substantial heterogeneity remained across these studies in xerostomia endpoint definitions (patient-reported, clinician-reported, and objective salivary function), assessment timing, dose modeling strategies (continuous or threshold-based), and statistical estimands (binary odds ratios, ordinal odds ratios, and regression coefficients), precluding quantitative synthesis (Supplementary Table 8).

#### 3.2.7 Certainty of evidence

The certainty of evidence was assessed for five organ–dose metric combinations (Supplementary Table 9), including contralateral parotid gland Dmean, bilateral parotid gland Dmean, ipsilateral parotid gland V30, contralateral submandibular gland Dmean, and oral cavity Dmean. Moderate-certainty evidence supported the associations of contralateral parotid gland Dmean and contralateral submandibular gland Dmean with xerostomia. Bilateral parotid gland Dmean and oral cavity Dmean were supported by low-certainty evidence, whereas ipsilateral parotid gland V30 remained supported by very-low-certainty evidence.

## 4 Discussion

We summarized 51 recent studies examining associations between post-radiation xerostomia and dosimetric parameters of organs at risk in patients with head and neck cancer. To our knowledge, only one prior systematic review addressing a similar topic was published in 2013 [12], primarily focusing on parotid gland mean dose in the earlier IMRT era.

Overall, the findings of the present review are broadly consistent with previous literature, supporting parotid gland mean dose as the most robust and consistently validated predictor of xerostomia. Contemporary studies continue to support the clinical relevance of limiting contralateral and bilateral parotid mean dose in modern IMRT-based radiotherapy. However, more recent evidence suggests that xerostomia risk is not solely determined by whole-parotid mean dose, but may also be influenced by dosimetric exposure of the submandibular glands, oral cavity, and functionally relevant parotid subregions. In parallel, NTCP modeling approaches have progressively evolved from single-organ toward multi-organ risk prediction frameworks.

### 4.1 Parotid gland dosimetry and xerostomia

The QUANTEC consensus previously proposed dose constraints of a mean dose <20 Gy to at least one parotid gland or < 25 Gy to both parotid glands; however, it did not explicitly differentiate between individual glands in terms of prioritization. In clinical practice, the contralateral parotid gland is the structure that is relatively easy to spare since it is often away from the high dose target areas. The studies summarized in this systematic review indicate that parotid mean dose remains the most frequently evaluated and consistently validated dosimetric predictor of xerostomia, particularly for the contralateral parotid gland. Multiple studies have repeatedly identified contralateral parotid Dmean as an independent predictor of xerostomia [14], [15], [16], with a marked increase in the incidence of moderate-to-severe late xerostomia observed when contralateral parotid Dmean exceeds approximately 24–26 Gy [14], [17], [18]. Conversely, maintaining contralateral parotid Dmean well below 20 Gy has been associated with a further reduction in early (3-month) severe salivary function impairment [19]. Based on the available evidence, the evidence synthesized in this review is consistent with a planning consideration of maintaining contralateral parotid Dmean ≤20 Gy to reduce the risk of early severe salivary dysfunction. In situations where this target is not achievable, maintaining contralateral parotid Dmean below approximately 24–26 Gy may be associated with a lower risk of moderate-to-severe late xerostomia.

For the bilateral parotid glands, available evidence indicates that a bilateral parotid Dmean ≥26 Gy is associated with a substantially increased risk of late xerostomia (HR ≈ 5.19) [20]. Overall, the included studies consistently support an average dose of approximately 26 Gy to both parotid glands (or to the total parotid gland volume) as a key baseline constraint for reducing the overall risk of clinically relevant xerostomia [20], [21], in line with previous QUANTEC recommendations. In contrast, for the ipsilateral parotid gland, dosimetric evaluation beyond mean dose appears to be particularly relevant. The moderate-dose volume fraction (V30) has emerged as an independent predictor of late xerostomia [16], [22], [23]. As the ipsilateral parotid gland is typically closer to the target volume and therefore more difficult to spare in terms of mean dose, V30 may provide a more practical representation of the residual functional gland volume. In clinical practice, a planning evaluation threshold of V30 < 50% is commonly applied, with a comparable risk cutoff (V30 ≈ 53.6%) reported in the literature [16]. Furthermore, in challenging cases where bilateral parotid mean dose constraints cannot be achieved, V30 has remained significantly associated with severe late xerostomia, suggesting that this parameter can offer a more direct characterization of the extent of functional gland volume receiving ≥30 Gy when mean dose sparing is limited [23].

In addition to whole-gland parotid dosimetry, recent studies have increasingly focused on parotid substructures-including the ducts, superficial lobes, and stem cell–rich regions (SCRs)-to explain the persistence of xerostomia despite compliance with conventional parotid dose constraints. These studies support the presence of functional heterogeneity within the parotid gland. Specifically, ductal dose and bilateral superficial lobe Dmean have both been reported to be closely associated with patient-reported xerostomia [24], [25], [26]. In addition, the relative contribution of SCR dose to xerostomia risk may exceed that of whole-parotid Dmean [10], [27]. However, the lack of standardized definitions and contouring protocols for parotid substructures limits cross-institutional reproducibility and workflow feasibility, and the current evidence-largely derived from retrospective studies with small sample sizes-has not yet translated into widely accepted clinical dose constraints.

### 4.2 Dosimetric associations in other salivary and oral structures

Mean dose to the contralateral SMG and the oral cavity (OC) also appears to provide additional information regarding xerostomia risk beyond the parotid glands. In line with the differential physiological roles of major salivary glands under stimulated and unstimulated conditions, dosimetric exposure of the submandibular glands and oral cavity may be particularly relevant for baseline and nighttime xerostomia. Within this physiological context, a LASSO-based NTCP model in nasopharyngeal cancer patients treated with helical tomotherapy identified contralateral SMG Dmean (for acute xerostomia) and OC Dmean (for late xerostomia) as major predictors, even when bilateral parotid mean dose constraints consistent with QUANTEC recommendations (≤25 Gy) were met [28]. Across the included studies, reported threshold values for contralateral SMG and OC mean dose varied to some extent; however, the direction of effect and the associated risk ranges were highly consistent, with risk emergence generally observed in the intermediate-to-high dose range (approximately 25–35 Gy) [8], [28], [29], [30]. Accordingly, in clinical treatment planning, once reasonable parotid dose sparing has been achieved, further reduction of contralateral SMG and OC mean dose-particularly avoidance of dose escalation into the ∼30 Gy and higher range-may help mitigate residual xerostomia burden, although this depends on the type of salivary flow to be maintained (stimulated vs. unstimulated flow).

### 4.3 Strength of evidence for dosimetric associations and planning thresholds

Moderate-certainty evidence supported the associations of contralateral parotid gland Dmean and contralateral submandibular gland Dmean with xerostomia (Supplementary Table 9). These findings strengthen the evidence supporting these two dosimetric parameters as clinically relevant predictors of radiation-induced xerostomia.

However, greater uncertainty remains when translating these dose–response associations into specific numerical planning thresholds. To facilitate clinical interpretation, recurrent planning thresholds identified from the narrative synthesis were therefore summarized and their threshold-specific certainty was evaluated separately (Supplementary Table 10). Threshold-specific certainty remained low or very low, reflecting the limited number of studies evaluating comparable cut-offs and the lack of independent external validation. These thresholds should therefore be regarded as pragmatic planning references rather than universally validated clinical dose constraints.

More broadly, both the identified dose–response associations and the proposed planning thresholds should be interpreted in the context of the considerable methodological and clinical heterogeneity across the included studies. Future studies using more standardized xerostomia definitions, assessment methods, dosimetric reporting, and follow-up schedules across diverse patient populations would facilitate quantitative evidence synthesis and strengthen the generalizability of clinically applicable dose constraints.

### 4.4 Heterogeneity in xerostomia assessment and timing

To assess xerostomia, the included studies employed a wide range of evaluation approaches (Fig. 2), encompassing clinician-reported assessments, patient-reported outcome measures, and objective evaluations. This heterogeneity in assessment methods poses a substantial challenge for cross-study comparison. In addition, as discussed above, differential contributions of individual salivary glands to xerostomia may result in distinct symptom profiles under daytime versus nighttime conditions and stimulated versus unstimulated states. Notably, among the assessment tools used in the included studies, the Groningen Radiotherapy-Induced Xerostomia Questionnaire (GRIX) was the only instrument that explicitly and systematically distinguished between daytime and nighttime xerostomia. However, only two of the 51 included studies applied GRIX to evaluate xerostomia, which limits the current evidence base for a systematic understanding of context-specific xerostomia symptoms. Furthermore, heterogeneity in the timing of xerostomia assessment across studies (Fig. 3) further compromises comparability of reported outcomes and may, to some extent, obscure differential roles of individual salivary gland structures in the development and persistence of xerostomia.

### 4.5 Implications for NTCP modeling and proton therapy selection

In the Netherlands, NTCP–based models are currently widely applied for patient selection in proton therapy, with the aim of predicting the risk of post-radiation xerostomia and estimating the potential benefit of proton therapy relative to photon-based radiotherapy [31], [32], [33]. The initial version of the National Indication Protocol for Proton Therapy (NIPP, version 1.0) primarily considered patient derived moderate-to-severe xerostomia (grade 2–3) at 6 months after radiotherapy as a single endpoint, with contralateral parotid mean dose serving as the key predictor [34]. In contrast, the updated NIPP version 2.2 introduced a systematic refinement of the model structure [35]. On the outcome level, xerostomia was stratified into grade ≥ 2 and grade ≥ 3 risk thresholds to more precisely differentiate clinically relevant severity levels. On the predictor level, the model was expanded from a single contralateral parotid mean dose to a multi-gland framework incorporating ipsilateral parotid mean dose, contralateral parotid mean dose, and bilateral submandibular gland mean dose. This evolution reflects a transition from a parotid-centered, uniform xerostomia model toward a multi-organ, multi-dimensional approach aimed at explaining inter-patient heterogeneity in xerostomia risk. These updates are consistent with the findings of the present review, namely that parotid dose remains a foundational predictor of xerostomia risk, while dosimetric parameters of the submandibular glands and other related structures provide important complementary information.

Recent phase III randomized trials have further strengthened the clinical evidence supporting proton therapy in head and neck cancer. The multicentre trial by Frank et al. [36] demonstrated non-inferior disease control together with lower rates of several high-grade toxicities following IMPT, whereas the TORPEdO trial [37] similarly demonstrated comparable oncological outcomes but did not demonstrate significant improvements in either co-primary endpoint, despite reduced doses to multiple organs at risk and fewer selected acute toxicities. Collectively, these studies suggest that although the magnitude of clinical benefit may vary across different endpoints, the dosimetric advantages of IMPT remain biologically and physically well founded. In particular, the finite range and absence of exit dose enable IMPT to substantially reduce the low-dose bath to surrounding normal tissues, including the contralateral parotid gland, compared with photon-based techniques [38], [39]**.** Given that contralateral parotid mean dose was identified in the present review as the most consistently supported predictor of radiation-induced xerostomia, these contemporary data further support the rationale for incorporating robust dose–response predictors into NTCP-guided proton therapy selection.

From a longer-term perspective, future development of NTCP modeling and proton therapy patient selection may benefit from the gradual incorporation of more context-specific outcome definitions-such as distinguishing between unstimulated and stimulated conditions, or daytime and nighttime xerostomia-as well as more refined structural and dosimetric parameters, including ipsilateral parotid V30, oral cavity mean dose, and stem cell–rich regions, provided that model robustness and generalizability are maintained.

### 4.6 Limitations

This study has several limitations. First, substantial heterogeneity was present among the included studies with respect to study design, xerostomia assessment methods and time points, the organs analyzed, and the dosimetric parameters reported. To facilitate structured evidence synthesis despite this heterogeneity, we developed a three-level evidence classification framework based on the interpretability and statistical robustness of the reported dose–response analyses. This framework was intended as a pragmatic methodological approach to distinguish studies according to the comparability of their reported evidence rather than as a formal evidence grading system. It also enabled the identification of studies potentially suitable for assessing the feasibility of quantitative synthesis. Nevertheless, this framework has not been formally validated and should therefore be interpreted as review-specific rather than universally applicable. Using this framework, we further evaluated the feasibility of quantitative synthesis for the most frequently reported dosimetric predictor (contralateral parotid mean dose) among studies meeting the predefined Level A criteria (Supplementary Table 8). Despite evaluating the same dosimetric predictor, substantial methodological heterogeneity remained in xerostomia endpoint definition, assessment timing, dose modeling strategy, and statistical estimands. Consequently, no clinically meaningful common effect estimate could be identified, precluding a formal meta-analysis.

Second, several potentially important clinical confounders were not explicitly accounted for in this review. Although both definitive and postoperative radiotherapy cohorts were eligible, surgery-related factors may influence xerostomia before radiotherapy and could act as residual confounders in xerostomia risk modeling. However, information on surgical variables was inconsistently reported across studies and therefore could not be systematically analyzed, and no sensitivity analyses stratified by treatment setting (e.g., postoperative versus definitive radiotherapy) were performed. Similarly, information regarding concurrent systemic therapy, including the use and type of concurrent or induction chemotherapy, was inconsistently reported across studies and therefore could not be systematically evaluated. This is particularly relevant because concurrent cisplatin-based chemoradiotherapy is associated with a higher risk of severe oral mucositis [40]**,** and treatment-related toxicity may represent an additional source of clinical confounding when evaluating xerostomia outcomes. Furthermore, subgroup analyses according to primary tumour site were not feasible because tumour subsite information was inconsistently reported or insufficiently detailed across the included studies. Given the substantial anatomical and treatment-planning differences among head and neck cancer subsites, the achievable degree of salivary gland sparing may differ considerably between tumour sites. Consequently, the inability to perform tumour-site-specific analyses may have introduced residual confounding and should be considered when interpreting the identified dose–response relationships.

Third, based on the applied risk-of-bias assessment tools (NOS, QUIPS, and RoB 2), only approximately one-third of the included studies (17/51) were rated as high quality. This indicates that some results may be subject to bias, potentially leading to over- or underestimation of effect sizes and reduced comparability and robustness of conclusions across studies.

Finally, non-English studies were excluded (*n* = 3), which may introduce language-restriction bias and limit the comprehensiveness of the evidence base. Publication bias also cannot be excluded, although a formal assessment was not feasible because of the substantial methodological heterogeneity and the absence of quantitative meta-analysis. Therefore, the available evidence should be interpreted with appropriate caution, as unpublished studies with null or negative findings may not be adequately represented. In addition, as the included literature consisted predominantly of retrospective cohort studies and lacked multicenter prospective validation, the generalizability of the present findings should be interpreted with caution.

### 4.7 Future research directions

Future research should focus on further standardization of study design and endpoint definitions, particularly with respect to the selection of xerostomia assessment instruments. Systematic incorporation of patient-reported outcome measures that distinguish between unstimulated and stimulated conditions, as well as between daytime and nighttime xerostomia, would be valuable. In addition, integration of objective salivary flow measurements (e.g., unstimulated and stimulated salivary flow rates) with gland-specific dosimetric analyses may help to more precisely delineate the relative contributions of different salivary gland structures to the development and persistence of xerostomia. Ultimately, the integration of multi-organ and multi-parameter information into NTCP models, supported by adequately powered, multicenter prospective studies with appropriate bias control, may provide a more robust evidence base for function-preserving, individualized radiotherapy decision-making.

Beyond conventional dose–volume predictors, radiomics has emerged as a promising approach for improving individualized prediction of radiation-induced xerostomia. By extracting high-dimensional imaging biomarkers that reflect patient-specific tissue characteristics and dynamic treatment-related changes, radiomics may capture biological information beyond that provided by conventional dose–volume histogram (DVH) metrics. Recent studies further suggest that longitudinal delta-radiomics and deep learning–based NTCP models can improve xerostomia prediction by leveraging temporal and three-dimensional imaging information, highlighting the potential of artificial intelligence–driven approaches for more accurate and personalized toxicity prediction. [41], [42], [43] In parallel, dosiomics may help overcome some of the inherent limitations of conventional dose–volume parameters by characterizing the spatial heterogeneity of three-dimensional dose distributions rather than reducing dose information to a limited number of summary metrics. Emerging evidence suggests that integrating dosiomics with radiomics and conventional clinical–dosimetric variables may further improve risk stratification for radiation-induced xerostomia. Rather than replacing established dose–volume predictors, these complementary high-dimensional approaches may enhance future NTCP models by incorporating biological and spatial information that is not captured by conventional DVH parameters alone. [44]

At the same time, radiotherapy strategies are increasingly shifting toward more individualized treatment approaches aimed at reducing treatment-related toxicity. Recent evidence supports risk-adapted elective nodal irradiation, including reduced elective dose or omission in appropriately selected patients, reflecting a shift away from routine bilateral neck irradiation for all patients. These approaches may substantially reduce unnecessary irradiation of the major salivary glands while maintaining oncological safety. [45] Treatment individualization is also increasingly guided by tumour biology. In particular, the favourable prognosis and enhanced radiosensitivity of HPV-associated oropharyngeal cancer have stimulated the development of de-escalation strategies, highlighting the importance of tailoring target volumes and dose prescriptions according to biological risk rather than applying uniform treatment approaches to all patients. [46] Ultimately, integrating advanced imaging biomarkers, spatial dose information, and individualized treatment strategies with conventional dose–volume predictors may facilitate the development of more robust NTCP models and further improve personalized prevention of radiation-induced xerostomia.

## 5 Conclusions

Overall, the findings of this systematic review are consistent with previous literature in supporting the central role of parotid mean dose in the prediction of xerostomia. Building on this foundation, the present review integrates more recent evidence and provides a comprehensive evaluation of dose–effect relationships between xerostomia and multiple organs at risk and their associated dosimetric parameters. In addition, the certainty of evidence for the principal organ–dose associations and recurrent planning thresholds was systematically evaluated to facilitate clinical interpretation.

Although considerable heterogeneity existed among the included studies in terms of study design, assessment methods, and evaluation time points, and overall study quality was variable, higher-quality studies demonstrated a consistent direction of effect. The strongest evidence supported contralateral parotid gland Dmean and contralateral submandibular gland Dmean as clinically relevant dosimetric predictors. Overall, these findings are consistent with current clinical practice and support a stratified consideration of contralateral parotid mean dose within the 20–26 Gy range. In addition, they suggest that, after achieving reasonable parotid dose sparing, additional dosimetric parameters of the submandibular glands and oral cavity may contribute to more refined risk assessment of long-term subjective xerostomia.

Nevertheless, given the heterogeneity and variable certainty of the available evidence, these findings should be interpreted with caution. At present, integrating this dosimetric information into function-preserving radiotherapy planning appears reasonable, although the evidence supporting specific numerical planning thresholds remains limited and requires further prospective validation. Future multicenter prospective studies employing standardized radiotherapy techniques, uniform xerostomia assessment instruments, and clearly defined assessment time points are needed to strengthen the evidence base and support continued refinement of xerostomia risk prediction models and clinical dose constraints.

## CRediT authorship contribution statement

**Jikai Zhou:** Writing – original draft, Visualization, Methodology, Investigation, Formal analysis, Data curation, Conceptualization. **Xuanhe Hou:** Writing – review & editing, Data curation. **Wenjie Liang:** Writing – review & editing, Data curation. **Frank Hoebers:** Writing – review & editing, Supervision. **Jolien Heukelom:** Writing – review & editing, Supervision. **Andre Dekker:** Writing – review & editing, Supervision. **Cecile Wolfs:** Writing – review & editing, Supervision. **Petros Kalendralis:** Writing – review & editing, Supervision, Project administration, Methodology, Conceptualization.

## Declaration of competing interest

The authors declare that they have no known competing financial interests or personal relationships that could have appeared to influence the work reported in this paper.

## Appendix A Supplementary data

Supplementary Table 10

Supplementary material 11 PRISMA 2020 checklist
