# Supplementary material for: Dose-volume predictors of post-radiation xerostomia in head and neck cancer: A systematic review
Source: Clin Transl Radiat Oncol. 2026 Sep 6;62:101282. doi: 10.1016/j.ctro.2026.101282 (PMC13579681; doi:10.1016/j.ctro.2026.101282)
Supplement: Supplementary file 1 [file mmc1.docx]

**Supplementary Table 1.** Detailed Search Strategies Across Databases

| **Concept** | **Search** |
| --- | --- |
| Combine  (pubmed)  (head & neck cancers AND radiotherapy AND xerostomia AND Radiation Dosage) | (((("Head and Neck Neoplasms"[MeSH Terms] OR (("Head"[Mesh] OR "head"[Title/Abstract] OR "Neck"[Mesh] OR "neck"[Title/Abstract] OR "oral"[Title/Abstract] OR "thyroid"[Title/Abstract] OR "esophag*"[tiab] OR "chin*"[tiab] OR "nose*"[tiab] OR "nasal"[tiab] OR "sinonasal*"[tiab] OR "Paranasal*"[tiab] OR "Hypophar*"[tiab] OR "Nasoph*"[tiab] OR "Oroph*"[tiab] OR "larynx"[tiab] or "trachea*"[tiab]) AND ("Neoplasms"[Mesh] OR "cancer*"[Title/Abstract] OR "neoplasm*"[Title/Abstract] OR "carcinoma*"[Title/Abstract] OR "tumo*"[Title/Abstract] OR "adeno*"[tiab] OR "malignan*"[tiab] OR "nodule*"[tiab] OR "melanom*"[tiab] OR "angiosarcoma*"[tiab] OR "sarcoma*"[tiab])))) AND (("radiotherapy"[MeSH Terms] OR "radiotherapy"[MeSH Subheading] OR "radiotherap*"[Title/Abstract] OR "chemoradi*"[tiab] OR "irradiati*"[tiab] OR "radioimmuno*"[tiab] OR "IMRT"[tiab] OR "radiat*"[tiab] OR "proton therap*"[tiab] OR "beam therap*"[tiab] OR "Photon Therap*"[tiab] OR "RT"[tiab] OR "CRT"[tiab]))) AND (("Xerostomia"[Mesh] OR "Asialia"[Title/Abstract] OR "xerostom*"[tiab] OR "dry mouth"[tiab:~3] OR "dryness mouth"[tiab:~3] OR "salivary gland function*"[tiab] OR "salivary gland hypofunction*"[tiab] OR "hyposaliv*"[tiab] OR "salivat*"[tiab]))) AND (("Radiation Dosage"[Mesh] OR "dose*"[tiab] OR "dosage*"[tiab] OR "dosimetr*"[tiab] OR "dosing"[tiab])) NOT ("case reports"[Publication Type] OR "editorial"[Publication Type] OR "review"[Publication Type] OR ("Animals"[MeSH Terms] NOT "Humans"[MeSH Terms]))) AND (2013:2024[pdat]) |
|  |  |
|  |  |
|  |  |
|  |  |
| Combine  (embase)  (head & neck cancers AND radiotherapy AND xerostomia AND Radiation dosage) | (exp “Head and Neck tumor”/ OR ((exp Head/ OR exp Neck/ OR ("head" OR "neck" OR "oral" OR "thyroid" OR “esophag*” OR “chin*” OR “nose*” OR “nasal” OR "sinonasal*" OR "Paranasal*" OR "Hypophar*" OR "Nasoph*" OR "Oroph*" OR “larynx” OR “trachea*”).ti,ab,kf.) AND (exp Neoplasm/ OR ("cancer*" OR "neoplasm*" OR "carcinoma*" OR "tumo*" OR “adeno*” OR “malignan*” OR “nodule*” OR “melanom*” OR “angiosarcoma*” OR “sarcoma*”).ti,ab,kf.))) and (exp radiotherapy/ or "radiotherapy".fs. or ("radiotherap*" or "chemoradi*" or "irradiati*" or "radioimmuno*" or "IMRT" or "radiat*" or "proton therap*" or "beam therap*" or "Photon Therap*" or "RT" or "CRT").ti,ab,kf.) and (exp Xerostomia/ or ("Asialia" or "xerostom*" or "salivary gland function*" or "salivary gland hypofunction*" or "hyposaliv*" or "salivat*" or (("dry" or "dryness") adj4 "mouth")).ti,ab,kf.) and (exp "Radiological Parameters"/ or ("dose*" or "dosage*" or "dosimetr*" or "dosing").ti,ab,kf.) NOT (("Conference Abstract" OR "Conference Paper" OR "Conference Review" OR "Review" OR "Editorial" OR "Letter").pt OR (exp Animal/ NOT (exp Human/))) |
|  |  |
|  |  |
|  |  |
|  |  |
| Combine  (wos)  (head & neck cancers AND radiotherapy AND xerostomia AND Radiation Dosage) | (TS=("head" OR "neck" OR "oral" OR "thyroid" OR “esophag*” OR “chin*” OR “nose*” OR “nasal” OR "sinonasal*" OR "Paranasal*" OR "Hypophar*" OR "Nasoph*" OR "Oroph*" OR “larynx” OR “trachea*”) AND TS=("cancer*" OR "neoplasm*" OR "carcinoma*" OR "tumo*" OR “adeno*” OR “malignan*” OR “nodule*” OR “melanom*” OR “angiosarcoma*” OR “sarcoma*”)) AND (TS=("radiotherap*" OR “chemoradi*” OR “irradiati*” OR “radioimmuno*” OR “IMRT” OR “radiat*” OR “proton therap*” OR “beam therap*” OR "Photon Therap*" OR "RT" OR "CRT")) AND (TS=(“Asialia” OR “xerostom*” OR “salivary gland function*” OR “salivary gland hypofunction*” OR “hyposaliv*” OR “salivat*” ) OR TS=(("dry" OR "dryness") NEAR/3 ("mouth"))) AND (TS=(“dose*” OR “dosage*” OR “dosimetr*” OR “dosing”)) NOT DT=(Review OR Book Review OR Database Review OR Letter OR Editorial Material) |
|  |  |
|  |  |
|  |  |
| Combine  (Cochrane)  (head & neck cancers AND radiotherapy AND xerostomia AND Radiation Dosage) | ((([mh “Head and Neck neoplasms”] OR ([mh Head] OR [mh Neck] OR head OR neck OR oral OR thyroid OR esophag* OR chin* OR nose* OR nasal* OR sinonasal* OR Paranasal* OR Hypophar* OR Nasoph* OR Oroph* OR larynx OR trachea*):ti,ab,kw) AND ([mh Neoplasms] OR (cancer* OR neoplasm* OR carcinoma* OR tumo* OR adeno* OR malignan* OR nodule* OR melanom* OR angiosarcoma* OR sarcoma*):ti,ab,kw)) AND ([mh Radiotherapy] OR [mh /RT] OR (radiotherap* OR chemoradi* OR irradiati* OR radioimmuno* OR IMRT OR radiat* OR proton NEXT therap* OR beam NEXT therap* OR Photon NEXT Therap* OR RT OR CRT):ti,ab,kw) AND ([mh Xerostomia] OR (Asialia OR xerostom* OR salivary NEXT gland NEXT function* OR salivary NEXT gland NEXT hypofunction* OR hyposaliv* OR salivat* OR ((dry OR dryness) NEAR/4 (mouth))):ti,ab,kw) AND ([mh "Radiation Dosage"] OR (dose* OR dosage* OR dosimetr* OR dosing):ti,ab,kw)) NOT ([mh Animals] NOT ([mh Humans])) |
|  |  |
|  |  |
|  |  |
|  |  |
