# Supplementary material for: Dose-volume predictors of post-radiation xerostomia in head and neck cancer: A systematic review
Source: Clin Transl Radiat Oncol. 2026 Sep 6;62:101282. doi: 10.1016/j.ctro.2026.101282 (PMC13579681; doi:10.1016/j.ctro.2026.101282)
Supplement: Supplementary file 2 [file mmc10.docx]

## Supplementary Table 10. GRADE-informed Certainty Assessment of Narrative Dosimetric Planning Thresholds

| **Proposed threshold** | **No. of direct threshold studies** | **Study limitations** | **Inconsistency** | **Indirectness** | **Imprecision** | **Overall certainty** |
| --- | --- | --- | --- | --- | --- | --- |
| Contralateral PG Dmean ≈20 Gy | 2 | Not serious | Not serious | Serious | Not serious | Low |
| Contralateral PG Dmean ≈24–26 Gy | 3 | Serious | Not serious | Serious | Not serious | Low |
| Bilateral PG Dmean ≈25–26 Gy | 2 | Not serious | Not serious | Serious | Not serious | Low |
| Ipsilateral PG V30 ≈50–54% | 2 | Very serious | Serious | Serious | Serious | Very low |
| Contralateral SMG Dmean ≈25–35 Gy | 2 | Serious | Serious | Serious | Serious | Very low |
| Oral cavity Dmean ≈30 Gy | 2 | Serious | Not serious | Very serious | Serious | Very low |

### Notes

Threshold-specific certainty was assessed separately from certainty of the overall dose–response association.

Continuous dose–response studies were considered indirect supportive evidence unless they directly evaluated the proposed numerical threshold.

Thresholds derived from sample-specific ROC analyses, Youden index optimization or other data-driven cut-point methods without independent validation were downgraded for study limitations and/or indirectness.

**Abbreviations:**
PG, parotid gland; SMG, submandibular gland; Dmean, mean dose; V30, percentage volume receiving at least 30 Gy.
