# Supplementary material for: Dose-volume predictors of post-radiation xerostomia in head and neck cancer: A systematic review
Source: Clin Transl Radiat Oncol. 2026 Sep 6;62:101282. doi: 10.1016/j.ctro.2026.101282 (PMC13579681; doi:10.1016/j.ctro.2026.101282)
Supplement: Supplementary file 4 [file mmc2.docx]

# Supplementary Table 2. Data Extraction Information for Included Studies

| **First Author (year)** | **Sample Size** | **Tumor types** | **RT technique** | **Chemotherapy** | **Timing of assessment** | **Xerostomia assessment** | **Dosimetric parameter** | **Cutoff** | **Relative effect** | **Statistical method level** |
| --- | --- | --- | --- | --- | --- | --- | --- | --- | --- | --- |
| Kaixuan Yang(2021) | 311 | NPC | IMRT; VMAT |  | ≥2 y post-RT | PRO EORTC QLQ-H&N35 | Contra PG Dmean | Contra PG Dmean<24.4Gy | Contra parotid Dmean(OR:1.002,95%CI:1.001-1.003,p < 0.001) | A |
| Minying Li(2022) | 365 | NPC | VMAT |  |  | CRO RTOG/EORTC | Left PG D50%;Right PG D50%;Right PG Dmean;OC Dmean | NR | / | B |
| İpek Pınar Aral(2024) | 31 | HNC | IMRT |  | 3 and 6 mo | CRO RTOG | Left PG Dmean | NR | / | C |
| Mona Arbab MD(2022) | 169 | HNC | IMRT | 0.78 | During RT | MDASI-HN;PROMIS | Contra PG Dmean;Contra PG V30;Ipsi SMG Dmean;OC Dmean | NR | / | C |
| Anney Tuo(2025) | 86 | OPC | IMRT | 0.87 | 3 and 12 mo | PRO EORTC QLQ-H&N35 | OC Dmean;OC V40;Contra SMG V30;Contra PG V15;FOM V20 | NR | / | C |
| Boning Cai(2016) | 80 | HNC | IMRT:HT+ RapidArc/VMAT(RA) | 1.0 | Acute withou specific timing | CRO RTOG Acute | Contra PG Dmean | NR | / | C |
| Kiattisa Sommat(2019) | 172 | NPC | IMRT | 1.0 | 3;12;24;36;48;60 mo | CRO RTOG acute;PRO EORTC QLQ-HN35 | Contra PG V60;Contra PG V20 | NR | / | B |
| Lisa Van den Bosch(2021) | 1145 | HNC | 3D-CRT; IMRT; VMAT | 0.33 | During RT; at 3, 6, 12, 18, and 24 mo post-RT | PRO EORTC QLQ-HN35;CRO CTCAE | ∑√Dmean (PGs);√Dmean (OC);ln(Dmean Lt+Rt BM);Dmean (SMGs) | NR | / | B |
| Xin-Ling Cai(2022) | 32 | NPC | IMRT | 1.0 | 6-12months | CRO RTOG late | Dmean, D50%, V26, V30, V33, V45, V20cc for PG-only vs. PG+APG | NR | / | B |
| Charles S Mayo(2023) | 964 | HNC | NR |  |  | CRO no detail | Contra SMG (Low-dose region):D25%;D75% ;Contra PG (Low-dose region):D50% | Submandibular gland (Low-dose region): D25% ≥ 41 Gy (p < 0.05) D75% ≤ 28.9 Gy (p < 0.05) Parotid gland (Low-dose region): D50% ≤ 12.0 Gy (p < 0.05) | / | B |
| Bhishamjit S Chera(2017) | 45 | OPC | IMRT | 1.0 | 6;12 mo | PRO CTCAE ≥2 | Combined Contra glands V15;V25;V55;Dmean | Combined contralateral glands 6 mo: V15 < 67%  V25 < 46% V55 < 16%  Dmean < 25 Gy  12 mo V15 < 68%  Dmean < 28 Gy | / | B |
| Xin-Bin Pan(2021) | 195 | NPC | IMRT | 0.9 | 12 mo | CRO RTOG/EORTC | Ipsi PG V30 | NR | / | B |
| Kanchan S Madhwapathy(2024) | 34 | HNC | IMRT | 0.91 | Base line;2 weeks;3 ,6 mo post-RT | CRO RTOG/EORTC +PRO EORTC QLQ-H&N35 | Bilat PGs Dmean;Bilat superficial PGs Dmean | NR | / | C |
| Daniel H Schanne(2024) | 99 | HNC | IMRT | 0.96 | During RT;3;6months;≥2years post-RT | CRO CTCAE | Contra PG Dmean;Contra parotid duct PRV Dmean | 6–12months:Intraglandular ducts Dmean < ~14 Gy Total ducts Dmean < ~12 Gy | Acute(SSX ≥G2):Contra parotid Dmean OR=1.14 (1.02–1.30, p=0.02);Weighted bilateral parotid Dmean OR=0.84 (95%CI 0.73–0.95, p<0.01);Contra parotid PRV Dmean OR=1.84 (1.07–3.58, p=0.03);Weighted bilateral parotid PRV DmeanPD OR=0.55 (0.30–0.92, p=0.02)。 | A |
| Mishra, H.(2023) | 62 | OCC | IMRT | 1.0 | During RT,3;6months;1;2;3years post-RT | CRO CTCAE | Contra PG Dmean ;Contra SMG Dmean | Contra PG Dmean > 26 Gy Contra SMG Dmean> 26 Gy | Contra parotid Dmean >26 Gy:HR 4.32(95%CI 1.03–18.05,p=0.045)。 Contra SMG Dmean >26 Gy:HR 6.41(95%CI 1.48–27.81,p=0.013)。 | A |
| Manal Ahmidouch(2024) | 38 | OPC | VMAT;HT |  | 6 and 12months | PRO CTCAE | Total parotid duct Dmean;Intraglandular parotid ducts Dmean | Total parotid duct Dmean< 14 Gy;Intraglandular parotid ducts Dmean< 12 Gy | / | B |
| Dirk Rades(2022) | 145 | HNC | 3D-CRT | 0.57 | peak grade within 6 weeks–24 mo | LENT-SOMA Subjective | Bilat PGs D40;D60;D80;Dmean | NR | / | C |
| David V Fried(2022) | 244 | OPC | IMRT |  | 6;12months post-RT | PRO-CTCAE | OC Dmean;Contra PG Dmean ;Contra SMG Dmean;FOM Dmean | NR | 6months: • OC Dmean:OR 1.064 /Gy(95% CI 1.004–1.129,p=0.038) ; • Contra parotid Dmean:OR 1.039 /Gy(1.002–1.078,p=0.039); • Floor of mouth Dmean:OR 1.042 /Gy(1.002–1.084,p=0.039)。 12months: •Contra SMG Dmean:OR 1.06 /Gy(1.028–1.088,p<0.01) | A |
| Huageng Huang(2022) | 90 | NPC | IMRT | 0.9 | Base line;3;6;12 mo post-RT | CRO RTOG/EORTC;XQ;LENT-SOMA (Analytic);USFR | Bilat PGs Dmean;Bilat PGs V30;Bilat superficial PGs Dmean;Bilat superficial PGs V30 | NR | / | C |
| Jianzhong Cao(2021) | 532 | OPC | IMRT vs IMPT |  | 3;6;12;24;＞24 mo post-RT | XQ | OC V25;V30;V35;V40;V45;V50;V55;V60;V65;V70 | NR | / | C |
| Wei Han(2017) | 61 | NPC | IMRT |  | During RT;3;6 mo post-RT | EORTC QLQ-C30 | Bilat SMGs Dmean;D50% | Bilateral SMGs Dmean 40.16 Gy;D50 36.38 Gy | / | B |
| Maria Thor(2017) | 121 | HNC | IMRT |  | 3;12months post-RT | LENT-SOMA (Analytic) | Contra PG Dmean ;Ipsi PG Dmean | Contra PG Dmean <20 Gy,Ipsi PG Dmean≈25 Gy。 | Adjusted β (per Gy): contra = 0.17; ipsi = 0.11 (no OR reported) | A |
| Tsair-Fwu Lee(2014) | 236 | HNC | IMRT |  | Baseline;During RT;3;12months post-RT | PRO EORTC QLQ-H&N35 | Contra PG Dmean ;Ipsi PG Dmean | NR | HNSCC-3 mo:Dmean-c OR 1.892(95%CI 1.724–2.075),Dmean-i OR 1.203(1.164–1.243);HNSCC-12 mo:Dmean-c OR 4.054(3.287–≈5),Dmean-i OR 1.430(1.330–1.539);NPC-3 mo:Dmean-c OR 1.244(1.195–1.295),Dmean-i OR 1.203(1.157–1.251);NPC-12 mo:Dmean-c OR 1.747(1.613–1.893),Dmean-i OR 1.712(1.585–1.850) | A |
| Sandeep Muzumder(2021) | 103 | HNC | IMRT | 0.68 | 6 mo;1 ;2 ;3 y | CRO CTCAE | Bilat PGs Dmean | Bilat PGs Dmean≥ 26 Gy | Bilateral parotid glands Dmean ≥ 26 Gy HR=5.19,95%CI 1.90–14.22,p=0.001 | A |
| Jiawei Zhou(2024) | 415 | NPC | IMRT | 0.91 | 12;24 mo post-RT | CRO RTOG/EORTC | Bilat PGs Dmean;Sublingual glands Dmean | NR | / | C |
| Eva Onjukka(2020) | 753 | HNC | IMRT;3D-CRT |  | 9;12;24 mo | modified RTOG/LENT-SOMA | Bilat PGs Dmean | NR | XERG≥3 Bilateral parotid Dmean HR 1.01, p=0.054 | A |
| Anna Cavallo(2021) | 263 | HNC | IMRT;VMAT | 1.0 | Baseline;During RT | CRO CTCAE | Bilat PGs D98%;OC EUD | NR | cPG D98% OR=1.09(95%CI 1.01–1.17),OC EUD OR=1.08(95%CI 1.02–1.15) | A |
| Athanasios Tzikas(2023) | 231 | OPC | IMRT;VMAT | 1.0 | Baseline;During RT; 6;12;18;24 mo post-RT | PRO-CTCAE;CRO CTCAE | Combined contra glands V50;Contra PG V45;Contra SMG V35; | PRO CTCAE:Combined contralateral glands Dmean < 22 Gy;V50 < 10%;Contra PG Dmean < 18 Gy;V45 < 10%;Contra SMG Dmean < 40 Gy;V35 < 60%。 CTCAE:Combined contralateral glands Dmean < 25 Gy;V10 < 80%;Contra PG Dmean < 20 Gy;V10 < 65%;Contra SMG Dmean < 55 Gy;V45 < 95%。 | / | B |
| Masahiro Inada(2022) | 74 | NPC | IMRT |  | 1;2;3 y post-RT | CRO CTCAE | Contra PG Dmean | Contra PG Dmean 25.8 Gy | / | B |
| T Bird(2017) | 186 | OPC | IMRT |  | Base line;During RT;3;6;12 mo post-RT | CRO RTOG/EORTC | Contra PG Dmean | NR | / | C |
| Hubert Szymon Gabryś(2017) | 153 | HNC | IMRT;HT |  | 6;12 mo | CRO CTCAE | Contra PG Dmean;Bilat PGs Dmean | NR | / | B |
| Roel J H M Steenbakkers(2022) | 102 | HNC | IMRT;VMAT |  | 6;12;24months post-RT | EORTC QLQ-H&N35 Q41;GRIX ;CRO CTCAE | Contra SCR Dmean | NR | Contra SCR Dmean GRIX 6months OR 1.06(95%CI1.01–1.12); 12months OR 1.11(95%CI 1.02–1.21); 24months OR 1.34(95%CI 1.12–1.59); CRO CTCAE(≥2) 12 mo:OR 1.11(95%CI 1.02–1.20); 24 mo: OR 1.20(95%CI 1.04–1.39) | A |
| Tsair-Fwu Lee(2015) | 67 | NPC | HT | 0.69 | 1;6 mo | PRO QLQ-H&N35 | Contra SMG Dmean;Ipsi SMG Dmean;OC Dmean | NR | 1 mo:Ipsi SMG Dmean OR=1.353(95%CI 1.285–1.424);Contra SMG Dmean OR=1.337(95%CI1.269–1.409);OC Dmean OR=1.762(95%CI1.568–1.979); 6 mo:OC Dmean OR=1.648(95%CI1.468–1.851)。 | A |
| Maria I van Rijn-Dekker(2023) | 1013 | HNC | 3D-CRT;IMRT;VMAT;IMPT |  | 6;12;24 mo post-RT | EORTC QLQ-H&N35 Q41;GRIX;CRO CTCAE | Contra parotid Dreg = Dmean(SCR) + r × Dmean(non-SCR) | NR | GRIX Q1: 12 mo:β=0.008 → OR/1 Gy ≈ 1.008 24 mo:β=0.005 → OR/1 Gy ≈ 1.005 GRIX Q3: 6 mo:β=0.014 → OR/1 Gy ≈ 1.014 24 mo:β=0.006 → OR/1 Gy ≈ 1.006  CTCAE ≥2 : 6 mo:β=0.015 → OR/1 Gy ≈ 1.015 12 mo:β=0.021 → OR/1 Gy ≈ 1.021 24 mo:β=0.030 → OR/1 Gy ≈ 1.030(≈1.162/5 Gy)。 | A |
| Xin-Bin Pan(2020) | 195 | NPC | IMRT | 0.9 | 3;6;12 mo post-RT | CRO RTOG/EORTC | Ipsi PG V25;V30;V35;V45;Contra PG Dmean | Ipsi PG V25 62.23%;V30 53.59%;V35 46.62%;V45 33.02%;Contra PG Dmean 39.63 Gy | OR(95%CI):CPG.Dmean 2.04 (1.16–3.61, p=0.014);IPG.V25 6.95 (1.83–26.42, p=0.004);IPG.V30 0.02 (0.01–0.36, p=0.008);IPG.V35 77.44 (5.06–1184.51, p=0.002);IPG.V45 0.19 (0.00–0.56, p=0.003) | A |
| David V Fried(2022) | 40 | OPC | IMRT |  | 6;12 mo post-RT | PRO-CTCAE;EORTC QLQ-H&N35 | Bilat parotid duct Dmean;Bilat PG Dmean;Contra SMG Dmean | NR | All patients: 6 mo OR = 1.37 (0.99–1.88, p = 0.06, n.s.); 12 mo OR = 1.62 (1.06–2.49, p = 0.03). CRT de-escalation: 6 mo OR = 1.71 (1.02–2.86, p = 0.04); 12 mo OR = 2.56 (0.86–7.63, p = 0.09, n.s.). | A |
| Reddy, Navaneeth(2022) | 100 | HNC | 3D-CRT;IMRT |  | Base line;During RT;1;3;6 mo post-RT | XQ | Ipsi PG Dmin;V30;V40;V45;V60 | NR | / | C |
| Feng Teng(2019) | 175 | HNC | HT; IMRT |  | 1;3;6;12;18 mo post-RT | Xerostomia Questionnaire(XQ);USFR;LENT-SOMA (Analytic) | Bilat PGs Dmean;Contra SMG Dmean;OC Dmean | PG-T 29.12 Gy;cSMG 29.29 Gy;OC 31.44 Gy。 | / | B |
| J. K. Kaae(2019) | 109 | HNC | IMRT;VMAT | 0.65 | ≥6 mo post-RT | EORTC QLQ-H&N35;DAHANCA | OC Dmean | NR | OCDmean OR 1.06(95%CI 1.01–1.12,p=0.03) | A |
| Peter G Hawkins(2018) | 252 | HNC | IMRT | 0.93 | 1;3;6;12;18;24;＞24 mo post-RT | XQ;HNQOL | Bilat PGs Dmean;Contra SMG Dmean;OC Dmean | NR | / | C |
| Moses Tam(2015) | 125 | OPC | IMRT | 0.99 | First questionnaire (9–24 mo post-RT start) | XQ;CRO CTCAE | Contra PG Dmean;Contra SMG Dmean;OC Dmean | NR | / | C |
| Vrinda Singla(2021) | 30 | HNC | IMRT |  | Baseline;3;6;12 mo post-RT | EORTC QLQ-HN35 | Contra PG Dmean | NR | / | B |
| Vedang Murthy(2018) | 90 | HNC | IMRT | 0.73 | Baseline;6;12;18;24;>24 mo post-RT | Dynamic 99mTc scintigraphy | Bilat SMGs Dmean | NR | / | B |
| Valerio Nardone(2018) | 78 | HNC | IMRT | 0.53 | 1;3;6;9;12;18;24 mo post-RT | CRO CTCAE | Ipsi PG V30 | NR | 12 mo:Ipsi parotid V30 OR 8.45 (95%CI 2.56–26.56) | A |
| Dirk Rades(2022) | 159 | HNC | IMRT;3D-CRT |  | peak/worst grade within 6 weeks–24 mo | LENT-SOMA·Subjective | Ipsi PG Dmean | NR | / | C |
| Adepitan A Owosho(2019) | 63 | HNC | IMRT |  | <6 mo;6–11months;12–24months | LENT-SOMA (Analytic) | Ipsi PG Dmena;Contra PG Dmean;Bilat PGs Dmean | NR | / | B |
| Helena C Bitz(2024) | 180 | HNC | 3D-CRT/IMRT/VMAT/HT | 0.7 | 3;6;9;12;18;24 mo,＞2 y | CRO CTCAE | Soft palate EQD2 Dmean | NR | Soft palate EQD2 Dmean OR 1.028 | A |
| Matthijs H Valstar(2021) | 723 | HNC | NR |  | 6;12;18;24 mo post-RT | EORTC QLQ-H&N35;CRO CTCAE | Tubarial glands Dmean | NR | Tubarial glands Dmean,CRO CTCAE ≥G2 12 mo:OR 1.019 /Gy(95%CI 1.005–1.034) 24 mo:OR 1.018 /Gy(95%CI 1.003–1.034) | A |
| Ciro Franzese(2015) | 102 | HNC | VMAT |  | 3;6;12 mo post-RT | CRO CTCAE | Contra PG D2/3V | NR | / | C |
| Megi Gjini(2022) | 63 | HNC | IMRT |  | ≥9 mo,last assessment | PRO EORTC QLQ-H&N35 | Contra PG Dmean;Contra PG stem cell PRV Dmean | NR | / | B |
| Israël Fortin(2014) | 119 | HNC | sw-IMRT vs HT | 1.0 | 6;12;24 mo post-RT | CRO CTCAE | Bilat PGs Dmean | Bilateral PGs Dmean＞25.5 Gy | / | B |

**Notes**
**Abbreviations:**

3D-CRT, three-dimensional conformal radiotherapy; BM, buccal mucosa; CTCAE, Common Terminology Criteria for Adverse Events; CRO, clinician-reported outcome; CPG, contralateral parotid gland; CRT, chemoradiotherapy; CTV, clinical target volume; Dmean, mean dose; Dmin, minimum dose; EORTC QLQ-H&N35, European Organisation for Research and Treatment of Cancer Quality of Life Questionnaire Head and Neck module; EQD2, equivalent dose in 2-Gy fractions; EUD, equivalent uniform dose; FOM, floor of mouth; GRIX, Groningen Radiotherapy-Induced Xerostomia scale; HNC, head and neck cancer; HT, helical tomotherapy; IMPT, intensity-modulated proton therapy; IMRT, intensity-modulated radiotherapy; IPG, ipsilateral parotid gland; LENT-SOMA, Late Effects in Normal Tissues – Subjective, Objective, Management and Analytic scale; NPC, nasopharyngeal carcinoma; OAR, organ at risk; OC, oral cavity; OPC, oropharyngeal carcinoma; PG, parotid gland; PRV, planning organ-at-risk volume; PRO, patient-reported outcome; PRO-CTCAE, patient-reported Common Terminology Criteria for Adverse Events; PTV, planning target volume; RA, RapidArc/VMAT; RTOG/EORTC, Radiation Therapy Oncology Group/European Organisation for Research and Treatment of Cancer toxicity criteria; SCR, stem-cell-rich region; SMG, submandibular gland; TG, tubarial gland region; USFR, unstimulated salivary flow rate; VMAT, volumetric-modulated arc therapy; Vx, percentage volume receiving ≥x Gy; XQ, Xerostomia Questionnaire.

**Definition notes:**
Xerostomia outcomes included clinician-reported toxicity (CTCAE; RTOG/EORTC; LENT-SOMA) and patient-reported measures (EORTC QLQ-H&N35; PRO-CTCAE; XQ; HNQOL).

**Dose parameters:**
Dose–volume metrics (Dmean, Dmin, Vx, D50, D98, EUD, EQD2) reflect standard radiotherapy dosimetry definitions.
