# Supplementary material for: Dose-volume predictors of post-radiation xerostomia in head and neck cancer: A systematic review
Source: Clin Transl Radiat Oncol. 2026 Sep 6;62:101282. doi: 10.1016/j.ctro.2026.101282 (PMC13579681; doi:10.1016/j.ctro.2026.101282)
Supplement: Supplementary file 5 [file mmc3.docx]

**Supplementary Table 3.** NOS Quality Assessment for Included Cohort Studies

| **First Author (year)** | **S1. Representativeness** | **S2. Non-exposed cohort** | **S3. Exposure ascertainment** | **S4. Baseline xerostomia excluded** | **C1. Main confounders adjusted** | **C2. Additional confounders** | **O1. Outcome assessment** | **O2. Follow-up duration** | **O3. Follow-up adequacy** | **Total (0–9)** | **Quality level** |
| --- | --- | --- | --- | --- | --- | --- | --- | --- | --- | --- | --- |
| Kaixuan Yang(2021) | 1 | 0 | 1 | 1 | 1 | 1 | 1 | 1 | 1 | 8 | High |
| Minying Li(2022) | 1 | 0 | 1 | 0 | 0 | 0 | 1 | 0 | 1 | 4 | Low |
| İpek Pınar Aral(2024) | 1 | 0 | 1 | 0 | 0 | 0 | 1 | 1 | 0 | 4 | Low |
| Mona Arbab MD(2022) | 1 | 0 | 1 | 0 | 0 | 0 | 1 | 1 | 1 | 5 | Moderate |
| Anney Tuo(2025) | 1 | 1 | 1 | 0 | 1 | 1 | 1 | 1 | 1 | 8 | High |
| Boning Cai(2016) | 1 | 1 | 1 | 0 | 0 | 0 | 1 | 1 | 1 | 6 | Moderate |
| Kiattisa Sommat(2019) | 1 | 0 | 1 | 0 | 1 | 1 | 1 | 1 | 1 | 7 | High |
| Lisa Van den Bosch(2021) | 1 | 0 | 1 | 0 | 1 | 0 | 1 | 1 | 1 | 6 | Moderate |
| Xin-Ling Cai(2022) | 0 | 0 | 1 | 1 | 0 | 0 | 1 | 0 | 1 | 4 | Low |
| Charles S Mayo(2023) | 1 | 0 | 1 | 0 | 0 | 0 | 0 | 0 | 1 | 3 | Low |
| Xin-Bin Pan(2021) | 1 | 0 | 1 | 0 | 0 | 0 | 1 | 1 | 1 | 5 | Moderate |
| Kanchan S Madhwapathy(2024) | 1 | 0 | 1 | 1 | 0 | 0 | 1 | 0 | 1 | 5 | Moderate |
| Daniel H Schanne(2024) | 1 | 0 | 1 | 0 | 0 | 0 | 1 | 1 | 1 | 5 | Moderate |
| Mishra, H.(2023) | 1 | 1 | 1 | 0 | 0 | 0 | 1 | 1 | 1 | 6 | Moderate |
| Manal Ahmidouch(2024) | 1 | 1 | 1 | 1 | 0 | 0 | 1 | 1 | 1 | 7 | High |
| Dirk Rades(2022) | 1 | 1 | 1 | 0 | 0 | 0 | 1 | 0 | 1 | 5 | Moderate |
| Jianzhong Cao(2021) | 1 | 1 | 1 | 0 | 1 | 1 | 1 | 1 | 0 | 7 | High |
| Wei Han(2017) | 1 | 0 | 1 | 0 | 0 | 0 | 1 | 0 | 1 | 4 | Low |
| Maria Thor(2017) | 1 | 1 | 1 | 1 | 0 | 0 | 1 | 1 | 1 | 7 | High |
| Tsair-Fwu Lee(2014) | 1 | 0 | 1 | 1 | 1 | 1 | 1 | 1 | 0 | 7 | High |
| Sandeep Muzumder(2021) | 1 | 1 | 1 | 1 | 0 | 0 | 1 | 1 | 0 | 6 | Moderate |
| Jiawei Zhou(2024) | 1 | 1 | 1 | 1 | 1 | 0 | 1 | 1 | 0 | 7 | High |
| Eva Onjukka(2020) | 1 | 0 | 1 | 0 | 1 | 1 | 1 | 1 | 0 | 6 | Moderate |
| Anna Cavallo(2021) | 1 | 0 | 1 | 1 | 1 | 1 | 1 | 1 | 0 | 7 | High |
| Hubert Szymon Gabryś(2017) | 1 | 0 | 1 | 1 | 0 | 0 | 1 | 1 | 0 | 5 | Moderate |
| Tsair-Fwu Lee(2015) | 1 | 0 | 1 | 1 | 1 | 1 | 1 | 0 | 1 | 7 | High |
| Maria I van Rijn-Dekker(2023) | 1 | 0 | 1 | 1 | 1 | 1 | 1 | 1 | 0 | 7 | High |
| Xin-Bin Pan(2020) | 1 | 1 | 1 | 1 | 1 | 0 | 1 | 1 | 1 | 8 | High |
| David V Fried(2022) | 1 | 1 | 1 | 1 | 1 | 0 | 1 | 1 | 1 | 8 | High |
| Reddy, Navaneeth(2022) | 1 | 1 | 1 | 1 | 1 | 0 | 1 | 0 | 0 | 6 | Moderate |
| Feng Teng(2019) | 1 | 0 | 1 | 1 | 1 | 1 | 1 | 1 | 1 | 8 | High |
| Moses Tam(2015) | 1 | 1 | 1 | 0 | 0 | 0 | 1 | 1 | 0 | 5 | Moderate |
| Vrinda Singla(2021) | 1 | 0 | 1 | 0 | 1 | 0 | 1 | 1 | 0 | 5 | Moderate |
| Valerio Nardone(2018) | 1 | 1 | 1 | 1 | 1 | 0 | 1 | 1 | 0 | 7 | High |
| Dirk Rades(2022) | 1 | 0 | 1 | 1 | 1 | 0 | 1 | 1 | 0 | 6 | Moderate |
| Adepitan A Owosho(2019) | 1 | 1 | 1 | 1 | 0 | 0 | 1 | 1 | 0 | 6 | Moderate |
| Helena C Bitz(2024) | 1 | 0 | 1 | 1 | 0 | 0 | 1 | 1 | 1 | 6 | Moderate |
| Matthijs H Valstar(2021) | 1 | 1 | 1 | 0 | 1 | 1 | 1 | 1 | 0 | 7 | High |
| Ciro Franzese(2015) | 1 | 0 | 1 | 0 | 0 | 0 | 1 | 1 | 1 | 5 | Moderate |
| Megi Gjini(2022) | 1 | 0 | 1 | 0 | 0 | 0 | 1 | 1 | 0 | 4 | Low |
| Israël Fortin(2014) | 1 | 1 | 1 | 1 | 1 | 0 | 1 | 1 | 1 | 8 | High |

**Notes**

1 = criterion fulfilled; 0 = criterion not fulfilled.

Overall quality classification: High (7–9 points), Moderate (5–6 points), Low (0–4 points).

NOS domains: Selection (S1–S4), Comparability (C1–C2), and Outcome (O1–O3).
