# Supplementary material for: Dose-volume predictors of post-radiation xerostomia in head and neck cancer: A systematic review
Source: Clin Transl Radiat Oncol. 2026 Sep 6;62:101282. doi: 10.1016/j.ctro.2026.101282 (PMC13579681; doi:10.1016/j.ctro.2026.101282)
Supplement: Supplementary file 6 [file mmc4.docx]

**Supplementary Table 4.** QUIPS Risk-of-Bias Assessment (Prognostic/Modelling Studies)

| **First author (year)** | **SP** | **SA** | **PFM** | **OM** | **CONF** | **STAT** | **Overall Risk** |
| --- | --- | --- | --- | --- | --- | --- | --- |
| Bhishamjit S Chera(2017) | Moderate risk | Moderate risk | Low risk | Low risk | Moderate risk | Moderate risk | Moderate |
| David V Fried(2022) | Moderate risk | Moderate risk | Low risk | Low risk | Moderate risk | Low risk | Moderate |
| Athanasios Tzikas(2023) | Moderate risk | Moderate risk | Low risk | Low risk | Moderate risk | Moderate risk | Moderate |
| Masahiro Inada(2022) | Moderate risk | Low risk | Low risk | Low risk | Moderate risk | Moderate risk | Moderate |
| T Bird(2017) | Moderate risk | Low risk | Low risk | Low risk | High risk | Moderate risk | High |
| J. K. Kaae(2019) | Moderate risk | Low risk | low risk | low risk | Moderate risk | Moderate | Moderate |
| Peter G Hawkins(2018) | Moderate risk | Moderate risk | low risk | low risk | Moderate risk | Low risk | Moderate |
| Vedang Murthy(2018) | Low risk | Moderate risk | low risk | low risk | High risk | Moderate risk | High |

**Notes**

**Abbreviations:** RoB = risk of bias; SP = Study participation; SA = Study attrition; PFM = Prognostic factor measurement; OM = Outcome measurement; CONF = Study confounding; STAT = Statistical analysis/reporting.

**The overall risk of bias was summarized using a predefined decision rule for this review:**

Low risk of bias: all domains rated low;

Moderate risk of bias: one or more domains rated as moderate risk, provided that no domains were rated as high risk.

High risk of bias: any domain rated high.
