# Supplementary material for: Dose-volume predictors of post-radiation xerostomia in head and neck cancer: A systematic review
Source: Clin Transl Radiat Oncol. 2026 Sep 6;62:101282. doi: 10.1016/j.ctro.2026.101282 (PMC13579681; doi:10.1016/j.ctro.2026.101282)
Supplement: Supplementary file 7 [file mmc5.docx]

**Supplementary Table 5.** RoB 2 Assessment (Randomized Controlled Trials)

| ID | RND | DEV | MISS | MEAS | SEL | Overall RoB |
| --- | --- | --- | --- | --- | --- | --- |
| Huageng Huang(2022) | Some concerns | Low risk | Some concerns | Low risk | Some concerns | Moderate risk |
| Roel J H M Steenbakkers(2022) | Low risk | Low risk | Some concerns | Low risk | Some concerns | Moderate risk |

**Notes**

**Abbreviations:** RoB = risk of bias; RND = Randomization process; DEV = Deviations from intended interventions; MISS = Missing outcome data; MEAS = Measurement of the outcome; SEL = Selection of the reported result.

The overall *risk of bias* (RoB) for each outcome was derived from domain-level ratings according to the Cochrane RoB 2 algorithm.
Classifications were defined as follows:
Low risk of bias: all domains rated low;
Moderate risk of bias: at least one domain rated “some concerns” and none rated high;
High risk of bias: any domain rated high.
