# Supplementary material for: Dose-volume predictors of post-radiation xerostomia in head and neck cancer: A systematic review
Source: Clin Transl Radiat Oncol. 2026 Sep 6;62:101282. doi: 10.1016/j.ctro.2026.101282 (PMC13579681; doi:10.1016/j.ctro.2026.101282)
Supplement: Supplementary file 8 [file mmc6.docx]

**Supplementary Table 6.** Summary of Study-Level Methodological Quality and Risk-of-Bias Assessments

| **Study ID** | **Author (Year, Journal)** | **Tool** | **Quality** |
| --- | --- | --- | --- |
| S1 | Yang (2021, *Aging*) | NOS | High |
| S2 | Li (2022, *BMC Oral Health*) | NOS | Low |
| S3 | Aral (2024, *Cureus*) | NOS | Low |
| S4 | Arbab (2022, *Head & Neck*) | NOS | Moderate |
| S5 | Tuo (2025, *Radiother Oncol*) | NOS | High |
| S6 | Cai (2016, *Int J Clin Exp Med*) | NOS | Moderate |
| S7 | Sommat (2019, *Radiother Oncol*) | NOS | High |
| S8 | Van den Bosch (2021, *Radiother Oncol*) | NOS | Moderate |
| S9 | Cai (2022, *Front Oncol*) | NOS | Low |
| S10 | Mayo (2023, *medRxiv* – preprint) | NOS | Low |
| S11 | Pan (2021, *Front Oncol*) | NOS | Moderate |
| S12 | Madhwapathy (2024, *J Cancer Res Ther*) | NOS | Moderate |
| S13 | Schanne (2024, *Radiation Oncology*) | NOS | Moderate |
| S14 | Mishra (2023, *J Clin Diagn Res*) | NOS | Moderate |
| S15 | Ahmidouch (2024, *Medical Physics*) | NOS | High |
| S16 | Rades (2022, *Anticancer Research*) | NOS | Moderate |
| S17 | Cao (2021, *Radiother Oncol*) | NOS | High |
| S18 | Han (2017, *Biomedical Research*) | NOS | Low |
| S19 | Thor (2017, *Radiother Oncol*) | NOS | High |
| S20 | Lee (2014, *Scientific Reports*) | NOS | High |
| S21 | Muzumder (2021, *J Cancer Res Ther*) | NOS | Moderate |
| S22 | Zhou (2024, *BMC Cancer*) | NOS | High |
| S23 | Onjukka (2020, *Front Oncol*) | NOS | Moderate |
| S24 | Cavallo (2021, *Cancers*) | NOS | High |
| S25 | Gabryś (2017, *Acta Oncologica*) | NOS | Moderate |
| S26 | Lee (2015, *Scientific Reports*) | NOS | High |
| S27 | van Rijn-Dekker (2023, *Int J Radiat Oncol Biol Phys*) | NOS | High |
| S28 | Pan (2020, *Aging*) | NOS | High |
| S29 | Fried (2022, *Radiother Oncol*) | NOS | High |
| S30 | Reddy (2022, *Cancer Res Stat Treat*) | NOS | Moderate |
| S31 | Teng (2019, *BioMed Res Int*) | NOS | High |
| S32 | Tam (2015, *Am J Clin Oncol*) | NOS | Moderate |
| S33 | Singla (2021, *J Carcinogenesis*) | NOS | Moderate |
| S34 | Nardone (2018, *Radiologia Medica*) | NOS | High |
| S35 | Rades (2022, *Anticancer Research*) | NOS | Moderate |
| S36 | Owosho (2019, *J Cranio-Maxillofac Surg*) | NOS | Moderate |
| S37 | Bitz (2024, *Radiation Oncology*) | NOS | Moderate |
| S38 | Valstar (2021, *Radiother Oncol*) | NOS | High |
| S39 | Franzese (2015, *Radiation Oncology*) | NOS | Moderate |
| S40 | Gjini (2022, *Acta Oncologica*) | NOS | Low |
| S41 | Fortin (2014, *Head & Neck*) | NOS | High |
| S42 | Chera (2017, *Int J Radiat Oncol Biol Phys*) | QUIPS | Moderate |
| S43 | Fried (2022, *Adv Radiat Oncol*) | QUIPS | Moderate |
| S44 | Tzikas (2023, *Physica Medica*) | QUIPS | Moderate |
| S45 | Inada (2022, *Radiation Oncology*) | QUIPS | Moderate |
| S46 | Bird (2017, *Clin Otolaryngol*) | QUIPS | Low |
| S47 | Kaae (2019, *Acta Oncologica*) | QUIPS | Moderate |
| S48 | Hawkins (2018, *Radiother Oncol*) | QUIPS | Moderate |
| S49 | Murthy (2018, *Radiother Oncol*) | QUIPS | Low |
| S50 | Huang (2022, *Radiother Oncol*) | RoB 2 | Moderate |
| S51 | Steenbakkers (2022, *Clin Cancer Res*) | RoB 2 | Moderate |

**Notes**

**Abbreviations:** NOS, Newcastle–Ottawa Scale; QUIPS, Quality In Prognostic Studies; RoB 2, Cochrane Risk of Bias 2 tool.
Quality categories reflect methodological quality (NOS) or risk of bias (QUIPS/RoB 2), harmonized for cross-study comparison
(High = high quality or low risk of bias; Moderate = moderate quality or some concerns; Low = low quality or high risk of bias).
