# Supplementary material for: Dose-volume predictors of post-radiation xerostomia in head and neck cancer: A systematic review
Source: Clin Transl Radiat Oncol. 2026 Sep 6;62:101282. doi: 10.1016/j.ctro.2026.101282 (PMC13579681; doi:10.1016/j.ctro.2026.101282)
Supplement: Supplementary file 9 [file mmc7.docx]

**Supplementary Table 7.** Summary of Reported Dosimetric Predictors of Post-Radiation Xerostomia

| **Organ at risk** | **Dosimetric parameters**  **(No. of studies)** | **No. of studies reporting this OAR** |
| --- | --- | --- |
| Parotid glands |  |  |
| Contralateral PG | Dmean (16); V15 (1); V20 (1); V30 (1); V45 (1); V60 (1); D50% (1); D2/3V (1) | 20 |
| Ipsilateral PG | Dmean (3); V25 (1); V30 (4); V35 (1); V40 (1); V45 (2); V60 (1); Dmin (1) | 8 |
| Bilateral PGs | Dmean (12); ∑√Dmean (1); Weighted Dmean (1); V30 (1); D40% (1); D60% (1); D80% (1); D98% (1) | 15 |
| Left PG | Dmean (1); D50% (1) | 2 |
| Right PG | Dmean | 1 |
| Submandibular glands |  |  |
| Contralateral SMG | Dmean (7); V30 (1); V35 (1); D25% (1); D50% (1); D75% (1) | 10 |
| Ipsilateral SMG | Dmean (2) | 2 |
| Bilateral SMGs | Dmean (2); D50% (1) | 2 |
| Oral cavity and related structures |  |  |
| Oral cavity (OC) | Dmean (9); √Dmean (1); V25 (1); V30 (1); V35 (1); V40 (2); V45 (1); V50 (1); V55 (1); V60 (1); V65 (1); V70 (1); EUD (1) | 12 |
| Floor of mouth (FOM) | Dmean (1); V20 (1) | 2 |
| Buccal mucosa (BM) | ln(Dmean Lt+Rt BM) | 1 |
| Parotid gland–related structures |  |  |
| APG | Dmean; D50%; V26; V30; V33; V45; V20cc for PG-only vs. PG+APG | 1 |
| Superficial PGs | Dmean (2); V30 (1) | 2 |
| Parotid duct | Contralateral parotid duct PRV Dmean (1); Total parotid duct Dmean (1); Intraglandular parotid ducts Dmean (1) | 2 |
| Contralateral SCR | Dmean (1); Dreg = Dmean(SCR) + r × Dmean(non-SCR) (1); Stem cell PRV Dmean (1) | 3 |
| Other structures |  |  |
| Combined contralateral glands | V15 (1); V25 (1); V50 (1); V55 (1); Dmean (1) | 2 |
| Bilateral sublingual glands | Dmean | 1 |
| Soft palate | EQD2 Dmean | 1 |
| Tubarial glands | Dmean | 1 |

**Notes**

Numbers in parentheses indicate the number of studies reporting each dosimetric parameter. “No. of studies reporting this OAR” refers to the number of unique, non-duplicated studies in which at least one dosimetric parameter for the corresponding organ at risk was reported.

For organs evaluated in multiple studies, different dosimetric parameters may originate from different subsets of studies; therefore, the sum of parameter-specific counts may exceed the total number of studies reported for that organ.

For organs evaluated in a single study, parameter-specific counts may be omitted when all listed parameters originate from the same study.

**Abbreviations:**

PG, parotid gland; SMG, submandibular gland; OC, oral cavity; FOM, floor of mouth; BM, buccal mucosa; SCR, stem cell region; APG, accessory parotid gland; Dmean, mean dose; D50%, D75%, etc., dose received by 50%, 75%, etc., of the volume; Vx, percentage of organ volume receiving ≥x Gy; EQD2, equivalent dose in 2-Gy fractions; EUD, equivalent uniform dose; PRV, planning organ at risk volume; Contra, contralateral; Ipsi, ipsilateral; Bilat, bilateral; Lt/Rt, left/right.
