# Supplementary material for: Dose-volume predictors of post-radiation xerostomia in head and neck cancer: A systematic review
Source: Clin Transl Radiat Oncol. 2026 Sep 6;62:101282. doi: 10.1016/j.ctro.2026.101282 (PMC13579681; doi:10.1016/j.ctro.2026.101282)
Supplement: Supplementary file 10 [file mmc8.docx]

**Supplementary Table 8.** Feasibility assessment for quantitative synthesis of Level A studies reporting contralateral parotid mean dose

| **First Author (year)** | **Xerostomia assessment** | **Assessment timing** | **Dose modelling** | **Statistical model** | **Effect estimate** |
| --- | --- | --- | --- | --- | --- |
| Kaixuan Yang(2021) | EORTC QLQ-H&N35 | ≥2 years | Continuous | Multivariable logistic regression | Adjusted OR |
| Daniel H Schanne(2024) | CTCAE | Acute | Continuous | Multivariable logistic regression | Adjusted OR |
| Mishra, H.(2023) | CTCAE | Late | Threshold (>26 Gy) | Multivariable logistic regression | Adjusted HR |
| David V Fried(2022) | PRO-CTCAE | 6 & 12 months | Continuous | Multivariable ordinal logistic regression | Ordinal OR |
| Maria Thor(2017) | Stimulated whole-mouth saliva flow | 3 months | Continuous | Multivariable regression | Regression coefficient (β) |
| Tsair-Fwu Lee(2014) | EORTC QLQ-H&N35 | 3 & 12 months | Continuous | Multivariable logistic regression | Adjusted OR |
| Xin-Bin Pan(2020) | RTOG/EORTC | 12 months | Threshold (39.63 Gy) | Multivariable logistic regression | Adjusted OR |

**Notes**

Only Level A studies were evaluated because they reported adjusted dose–response models and therefore represented the only studies potentially eligible for quantitative synthesis.

**Abbreviations:**

CTCAE, Common Terminology Criteria for Adverse Events; PRO, patient-reported outcome; QLQ-H&N35, European Organisation for Research and Treatment of Cancer Quality of Life Questionnaire–Head and Neck Module; OR, odds ratio; HR, hazard ratio.
