# Supplementary material for: Dose-volume predictors of post-radiation xerostomia in head and neck cancer: A systematic review
Source: Clin Transl Radiat Oncol. 2026 Sep 6;62:101282. doi: 10.1016/j.ctro.2026.101282 (PMC13579681; doi:10.1016/j.ctro.2026.101282)
Supplement: Supplementary file 11 [file mmc9.docx]

## Supplementary Table 9. GRADE Assessment of Certainty of Evidence for Principal Organ-at-risk Dosimetric Parameters Associated with Post-radiotherapy Xerostomia

| **Principal dosimetric parameter** | **No. of independent studies** | **Study limitations** | **Inconsistency** | **Indirectness** | **Imprecision** | **Other considerations** | **Overall certainty** |
| --- | --- | --- | --- | --- | --- | --- | --- |
| Contralateral PG Dmean | 16 | Not serious | Not serious | Not serious | Not serious | Dose–response gradient | Moderate |
| Bilateral PG Dmean | 12 | Not serious | Not serious | Not serious | Not serious | None | Low |
| Ipsilateral PG V30 | 4 | Serious | Serious | Not serious | Serious | None | Very low |
| Contralateral SMG Dmean | 7 | Not serious | Not serious | Not serious | Not serious | Dose–response gradient | Moderate |
| Oral cavity Dmean | 9 | Not serious | Not serious | Serious | Not serious | Dose–response gradient | Low |

### Notes

Observational evidence initially started at low certainty according to the GRADE framework and was subsequently downgraded or upgraded according to study limitations, inconsistency, indirectness, imprecision and other considerations.

Study limitations reflected overall methodological quality (NOS, QUIPS and RoB2), risk of bias beyond study design, residual confounding, and analytical limitations.

Other considerations primarily reflected the presence or absence of a credible dose–response gradient across independent studies.

Formal GRADE assessment was restricted to organ–dose metric combinations evaluated in at least four independent studies with sufficiently comparable anatomical definitions and dosimetric parameters.

**Abbreviations:**
PG, parotid gland; SMG, submandibular gland; Dmean, mean dose; V30, percentage volume receiving at least 30 Gy.
